# On the Interaction between 1D Materials and Living Cells

**DOI:** 10.3390/jfb11020040

**Published:** 2020-06-10

**Authors:** Giuseppe Arrabito, Yana Aleeva, Vittorio Ferrara, Giuseppe Prestopino, Clara Chiappara, Bruno Pignataro

**Affiliations:** 1 Dipartimento di Fisica e Chimica—Emilio Segrè, University of Palermo, Viale delle Scienze, Ed.17, 90128 Palermo, Italy; bruno.pignataro@unipa.it; 2 INSTM UdR Palermo, Viale delle Scienze, Ed.17, 90128 Palermo, Italy; yana.aleeva@unipa.it (Y.A.); chiapparaclara@gmail.com (C.C.); 3 Dipartimento di Scienze Chimiche, Università di Catania, Viale Andrea Doria 6, 95125 Catania, Italy; v.ferrara@studium.unict.it; 4 Dipartimento di Ingegneria Industriale, Università di Roma “Tor Vergata”, Via del Politecnico 1, I-00133 Roma, Italy; giuseppe.prestopino@uniroma2.it

**Keywords:** 1D materials, biointerface, CNTs, polymers, bioelectronics, regenerative medicine, photocatalysis

## Abstract

One-dimensional (1D) materials allow for cutting-edge applications in biology, such as single-cell bioelectronics investigations, stimulation of the cellular membrane or the cytosol, cellular capture, tissue regeneration, antibacterial action, traction force investigation, and cellular lysis among others. The extraordinary development of this research field in the last ten years has been promoted by the possibility to engineer new classes of biointerfaces that integrate 1D materials as tools to trigger reconfigurable stimuli/probes at the sub-cellular resolution, mimicking the in vivo protein fibres organization of the extracellular matrix. After a brief overview of the theoretical models relevant for a quantitative description of the 1D material/cell interface, this work offers an unprecedented review of 1D nano- and microscale materials (inorganic, organic, biomolecular) explored so far in this vibrant research field, highlighting their emerging biological applications. The correlation between each 1D material chemistry and the resulting biological response is investigated, allowing to emphasize the advantages and the issues that each class presents. Finally, current challenges and future perspectives are discussed.

## 1. Introduction

The monitoring and manipulation of living cells by sub-cellular sized interfaces within platforms that mimic in vivo conditions represent an emerging field of research in materials science. These knowledge-platforms enable the measurement of cellular activities even at the single-cell resolution [1,2,3,4,5], allowing to regulate differentiation fate [6], producing intracellular cascades, and stimulating cellular communication across multiple length scales [7]. In this context, a fundamental role is played by the engineering of material interfaces, to achieve efficient coupling with cellular systems to recapitulate the in vivo scenario into a cell culture dish. Indeed, their physical and chemical properties strongly determine the ultimate behaviour of cells at the material–cell interface.

A typical classification based on their geometry and dimensions allows dividing materials into four groups, namely 0D, 1D, 2D, and 3D materials [8]. Briefly, 0D materials are those confined in all the three dimensions showing an almost spherical shape (cluster, dots, micelles, etc.). These are traditionally defined as 0D nanomaterials when their diameter is smaller than 100 nm. Further, 1D materials are characterized by a large length/diameter ratio (wires, tubes, etc.). If their diameter is smaller than the limit of 100 nm [9], they are usually defined as 1D nanomaterials. Differently, 2D materials feature a high diameter/thickness ratio (layers, films, sheets, etc.), and are conventionally defined as 2D nanomaterials if their thickness is smaller than 100 nm. Finally, 3D materials are characterized by all dimensions in the micrometre or larger scale. In principle, 3D materials are the most similar systems to the extracellular matrix, i.e., the complex network of rodlike structures composed of biomacromolecules (collagen, laminin, glycoproteins, etc.) that support cells in vivo [10]. This matrix is mainly constituted by protein fibres that self-assemble and polymerize into fibrils, ultimately resulting in protein networks that finely regulate cell adhesion and the intracellular diffusion of molecules in the extracellular domains.

However, the recapitulation of the 3D extracellular matrix in vitro is a challenging task, given its extreme complexity. Apart from its relevant role in chemical signaling, the extracellular matrix can also induce physical or mechanical stimuli, regulating cellular geometry, and ultimately their fate [11]. Researches have shown some simplified in vitro models able to mimic the extracellular matrix, constituted by mechanotunable 2D/3D microenvironments [12], collagen fibres leveraged for studies of cellular migration [13] or elastic scaffold produced by nanofibres [14]. In this scenario, 1D materials offer a wide and chemically versatile plethora of tools mimicking biological conditions in a synthetic platform, offering a high surface/volume ratio interface to evaluate and/or stimulate a cell, in an environment that recreates the extracellular matrix [15]. The favourable mechanic and electrical properties [16] have been leveraged for recording electrical cellular activities [17], exerting forces, and measuring enzymatic activities [18], membrane poration [19], and recently also directing stem cell behaviours in their development and regeneration [20,21].

**Figure 1:**
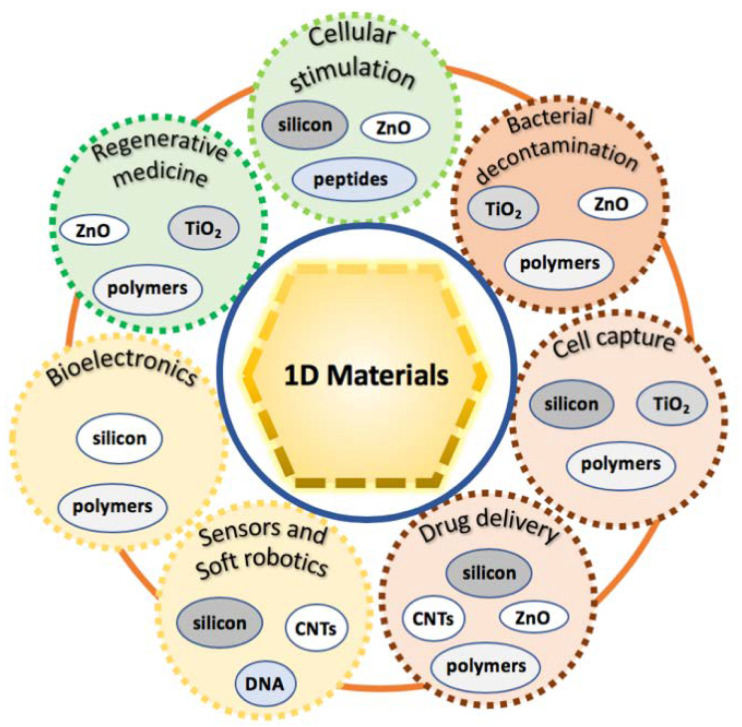
Main applications of 1D materials in life-sciences applications.

The present review offers an updated overview of the major classes of 1D materials (e.g., silicon, metal oxides, polymers, deoxyribonucleic acid (DNA), and peptides 1D nanostructures) for engineering biointerfaces, resulting in a useful correlation between the physicochemical properties of the investigated interfaces with cells, and the possible sets of available stimuli, by critically considering the effects of materials size and aspect ratio. As previously mentioned, 1D materials can have lateral sizes on the submicron-scale (>100 nm) or the nano-scale (<100 nm)—in the latter case, they are named as 1D nanomaterials. In turn, a further classification commonly used in literature for 1D nanomaterials considers their geometrical shapes/features, by adopting the terms nanocolumns (NCs), nanofibres (NFs), nanoneedles (NNs), nanopillars (NPLs), nanorods (NRs), nanotubes (NTs), and nanowires (NWs). Through this review, the same definitions will be used. The review provides a brief introduction (Section 2) to the theoretical models for the study of the cells/1D materials interfaces. The major classes of 1D materials are discussed in different sections: silicon (Section 3), titanium dioxide (Section 4), zinc oxide (Section 5), carbon nanotubes (Section 6), polymers (Section 7), and finally, DNA and peptides (Section 8). The synthesis, functionalization, and fundamental properties of these materials are briefly reported, and their cutting-edge applications in cellular biology are summarized, including electrical recording, cellular viability, adhesion, differentiation, enzymatic activity assay, differentiation switch, stem cell fate modulation, and antibacterial activity. Different applications will be highlighted through the review, namely regenerative medicine, cellular stimulation, bacterial decontamination, cellular capture, drug delivery, sensors, soft robotics and bioelectronics (see Figure 1), also permitting to find the possible classes of materials that can be better adapted for each of them. Each class of material is discussed by reporting examples of interfacing their structures with cellular systems, with the main aim of demonstrating the role of each specific 1D material in modulating the cellular response, finally shedding light on the challenges and the opportunities of this research field (Section 9).

## 2. Cells at the Interface with 1D Materials

In general, the mechanisms that define the cellular uptake of materials from the solution phase have been well investigated and can be divided in receptor-mediated endocytosis, pinocytosis, and phagocytosis (see the review from ref. [22]). On the contrary, the mechanism of a cell settling on an array of a 1D material (e.g., an NW array) at a solid interface is still not completely clear. For this reason, it is not possible to provide general principles governing the material–cell interaction, owing to unavoidable differences between different cells types (e.g., cancer cells, stem cells, bacteria, etc.). It is known from adult stem cells that the set of mechanical signals deriving from solid supported 1D materials regulates core components within the Hippo signalling cascade, including Yes-associated protein (YAP) and transcriptional coactivators with PDZ-binding motif (TAZ) [23] whose cross-talk signalling can determine the cell behaviour and fate [24]. As a result, the physico-chemical characteristics of the biointerface play a crucial role in directing the fate of the cellular system. Tailored biomaterials with specific bio-inspired structures can mimic the topographic landscape of the physiological microenvironment, i.e., the stem cell niche [25]. In this regard, the method of choice for surface structuring depends on the chosen material and pattern, taking into consideration the cues triggered by ligand/receptor binding and micro- or nano-topography. Notwithstanding the complexity of the problem, some physical models permit to quantify the interaction between cells and 1D materials, by taking into account differences in nanostructure size and arrangement [26,27,28]. Indeed, the extension of the membrane into the space between the nanostructures can be related to the increase of the interface area and its adhesion. Accordingly, it is possible to estimate the membrane deformation of a cell settling for instance into an array of NWs considering their density, length, and width [26]. This model permits to obtain the ΔG(bottom-top), which is the difference in the free energy between the states in which a cell is at top of the NWs tips and the scenario in which it is deformed and contacts the flat substrate underlying the NWs. If the case in which ΔG(bottom-top) > 0, the “top” settling is energetically favourable. For a single NW interface characterized by *x* NWs μm^–2^, having radius *r* and length *l*, considering a 1 μm^2^ area, the ΔG(bottom-top) is expressed as:

ΔG(bottom-top) = −*w* (1 μm^2^ + *x*2π*rl* − *x*π*r*^2^) + *σx*2π*rl* + *κx*π*r*^−1^ (1)

where *w*, *σ*, and *κ* denote the cell-related parameters (all expressed as [N·m^−1^]), namely the specific energy of adhesion per unit area, the surface tension, and the bending modulus, respectively. The same theoretical considerations can be employed to understand the physics behind the enhancement of cellular capturing on nanostructured arrays unlike flat planar surfaces, deriving from an equilibrium between the membrane adhesion and deformation energy [28,29]. Following the results of Zhou et al. [28], the adhesion-triggered modification of the free energy takes into account adhesion, bending, and stretching and it can be written as:

(2) $$\Delta E=-\int_{0}^{S_{ad}}\gamma dA+\int_{0}^{S_{bend}}\left[\frac{\kappa }{2}{\left(c_{1}+c_{2}-c_{0}\right)}^{2}\right]dA+\frac{1}{2}\lambda \frac{\Delta S^{2}}{S_{0}}$$

where γ [N·m^−1^] is the cell membrane/surface adhesion energy per unit area, *S_ad_* [m^2^] is the cell membrane/surface adhesion area, *k* [N·m^−1^] is the membrane curving modulus, *c*_1_ [m^−1^] and *c*_2_ [m^−1^] are two main membrane curvatures, *S_bend_* [m^2^] is the area of the curving membrane, and *λ* [N·m^−1^] is the membrane stretching modulus. $\Delta S=S-S_{0}$ and *S*_0_ are, respectively, the change in membrane interface and the initial total membrane interface during the adhesion. The first term in Equation (2) indicates the adhesion energy, the second term is the bending energy [30], and the third is the stretching free energy penalty [31]. From Equation (2), it results that patterned surfaces with high roughness parameter (the ratio between the total interfacial area between a cell and the nanostructures, and the projection area of an adhered cell from the top view) enhance cellular capture in comparison to less rough surfaces, because of the significant increase of the cell–nanostructures contact area. 

## 3. Silicon-Based 1D Nanomaterials

Silicon NWs and NTs have been widely demonstrated as key materials to build-up functional interfaces for bioelectronics [5,7,32,33,34,35,36,37], allowing for cutting-edge applications in subcellular biointerfaces [37,38]. The first application of Si NWs in bioelectronics emerged in 2006 by Patolsky and co-workers, in the form of open gate Si NW field-effect transistors (FETs) to record extracellular events from rat neurons [39]. In principle, silicon 1D nanomaterials can be produced by top-down approaches [40] or bottom-up strategies [35]. Among the bottom-up strategies, the nanocluster-catalysed vapour-liquid-solid (VLS) approach [41] is very popular given its simplicity and versatility. More recently, Si NWs have been synthesized by the laser ablation technique to obtain nanosized catalyst clusters facilitating VLS growth of high-quality Si and Ge NWs [42,43].

### Novel Applications in Drug Delivery and Cell Fate Regulation

**Figure 2:**
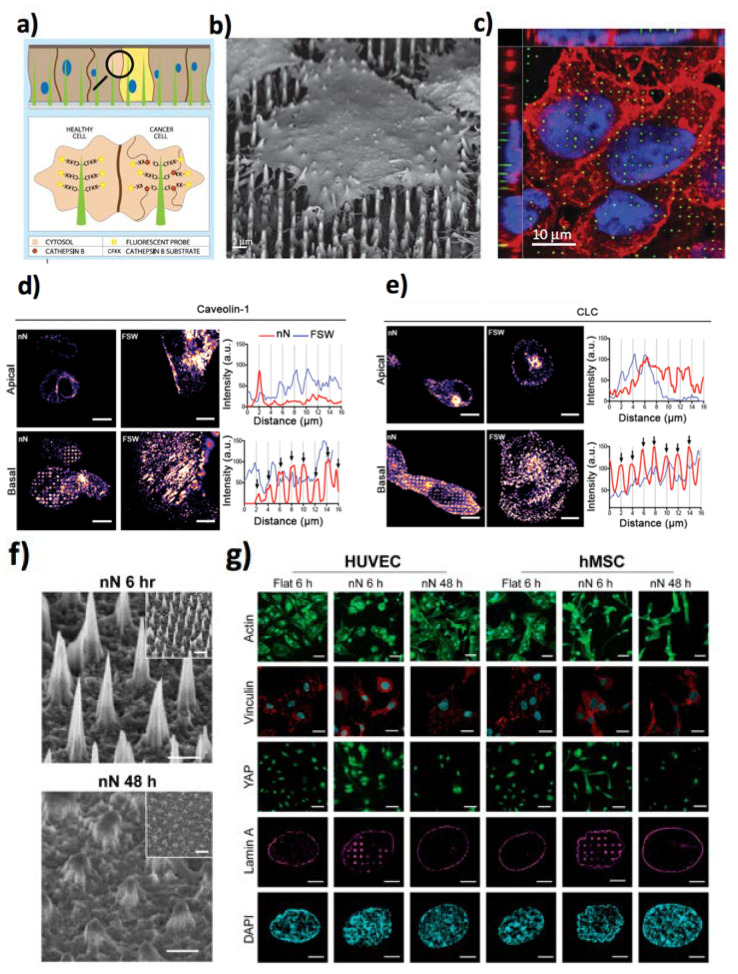

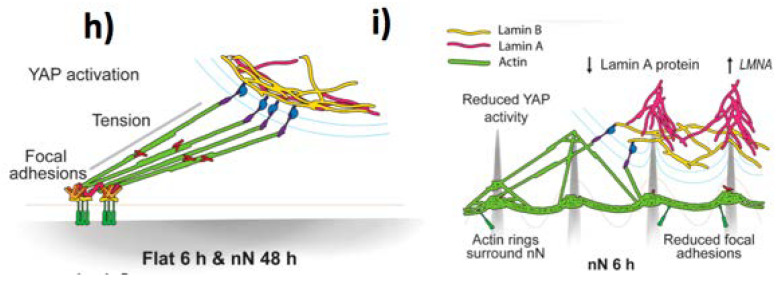
Si NWs-based single-cell stimulation and mechanosensing. (**a**) An NW-based sensor for cathepsin, relying on the CTSB cleaving its CFKK peptide substrate, freeing the linked TAMRA fluorescent probe. (**b**) Scanning electron microscopy and (**c**) laser scanning confocal fluorescence microscopy image of a cell onto NWs. NWs in green, cell membrane in red, nucleus in blue. Reproduced from ref. [48] under the terms of the Creative Commons Attribution license (CC BY). (**d**) Fluorescence confocal microscopy images of cell scaffolding protein Caveolin-1 and (**e**) clathrin (CLC) protein accumulation after 6 h in the membrane of hMSCs cultured on Si NWs. Scale bars are equal to 10 µm. Reproduced from ref. [49] under the terms of the Creative Commons Attribution license (CC BY). (**f**) Si NNs degradation after 48 h in cell culture. Scale bars are 1 μm, and 2 μm in the inset. (**g**) Restoration of cell phenotype on degraded NNs as compared to flat control substrates at 6 h. Cellular actin cytoskeleton (green: phalloidin, scale bars are 50 μm), dense staining of vinculin-rich focal adhesions (red: vinculin, cyan: DAPI, scale bars are 25 μm), nuclear localization of YAP (green, scale bars are 50 μm), and an unmodified nucleus (magenta: lamin A, cyan: DAPI, scale bars are 5 μm). (**h**) Intracellular tension on flat surfaces due to focal adhesion formation, yielding YAP nuclear localization. (**i**) Decreased focal adhesion of cells onto NNs triggers the generation of actin ring, and nuclear confinement of lamin A and B. Adapted with permission from ref. [54] (https://pubs.acs.org/doi/10.1021/acsnano.8b06998), Copyright (2019) American Chemical Society, further permissions related to the material excerpted should be directed to the ACS.

Si NWs have found applications for payload delivery, thanks to their permeability to the cellular membrane [44]. In 2016, Zimmerman et al. studied the cellular uptake of freestanding Si NWs [45] on human umbilical vein endothelial cell (HUVEC) and human aortic smooth muscle cell cultures. Andolfi et al. showed that high aspect ratio Si NWs control the mouse embryonic fibroblasts adhesion and cytoskeleton organization [46] by employing single-cell force spectroscopy to measure the adhesion strength down to pN resolution. In contrast to flat surfaces, the cell morphology and the organization of its cytoskeleton showed striking differences, since cells on Si NWs had a high number of filopodia and decreased mobility, along with a decreased proliferation after 36 h. Chiappini et al. [47] employed biodegradable Si NNs (having a 5 µm height, 1 µm pitch and <100 nm tips) for intracellular delivering of DNA and small interfering ribonucleic acid (siRNA). The NNs were leveraged to transfect in vivo the vascular endothelial growth factor (VEGF-165) gene, triggering neovascularization and localized blood perfusion in the target region of the muscle. The same kind of Si NNs were used to map the cytosolic cysteine protease Cathepsin B (CTSB) enzymatic activity in mucosa of the human esophagus [48]. NNs interfaced the cytosol of cells where active CTSB causes the cleavage of its Cathepsin B peptide substrate (CFKK), releasing the linked carboxytetramethylrhodamine fluorescent probe in the cell cytosol. The higher the CTSB activity, the higher was the fluorescence signal in the cytosol (Figure 2a–c). 

Recently, porous Si NNs [49] have shown the possibility to induce endocytosis, and deliver siRNAs into human mesenchymal stem cells (hMSCs). The NNs favoured the uptake of endocytosis biomolecular markers, along with micropinocytosis (Figure 2d,e). The Si NWs-induced cues permit to regulate the hMSCs fate [50]. Spherical morphology was observed for the hMSCs cultured on Si NWs and a 10% cell viability loss compared to those grown on flat Si supports. The shortest Si NWs triggered a higher expression of the osteogenic gene of type I collagen and runt-correspondent transcription factor 2 in comparison to the longer Si NWs and 2D flat Si. As another important example, Si NPLs arrays with diameters of 40–200 nm, obtained by the block copolymer colloids self-assembly, were used as efficient biointerfaces for differentiating hMSCs [51]. Si NWs have also found important applications in the field of neural stem cells (NSCs). Indeed, they can favour the differentiation of NSCs in neurons [52]. Kim et al. have shown the possibility to create strong contact between neurons and silicon nanocolumns (NCs) [53], retaining cellular integrity and the generation of neurites. To target mechanoresponsive elements inside HUVEC and hMSC cells, Hansel et al. [54] synthesized mesoporous Si NN arrays interfaces. They observed that NNs reduced the number of focal adhesions, decreasing the cytoskeletal stress, and remodelling the nuclear envelope at the contact sites (Figure 2f–i). 

**Figure 3:**
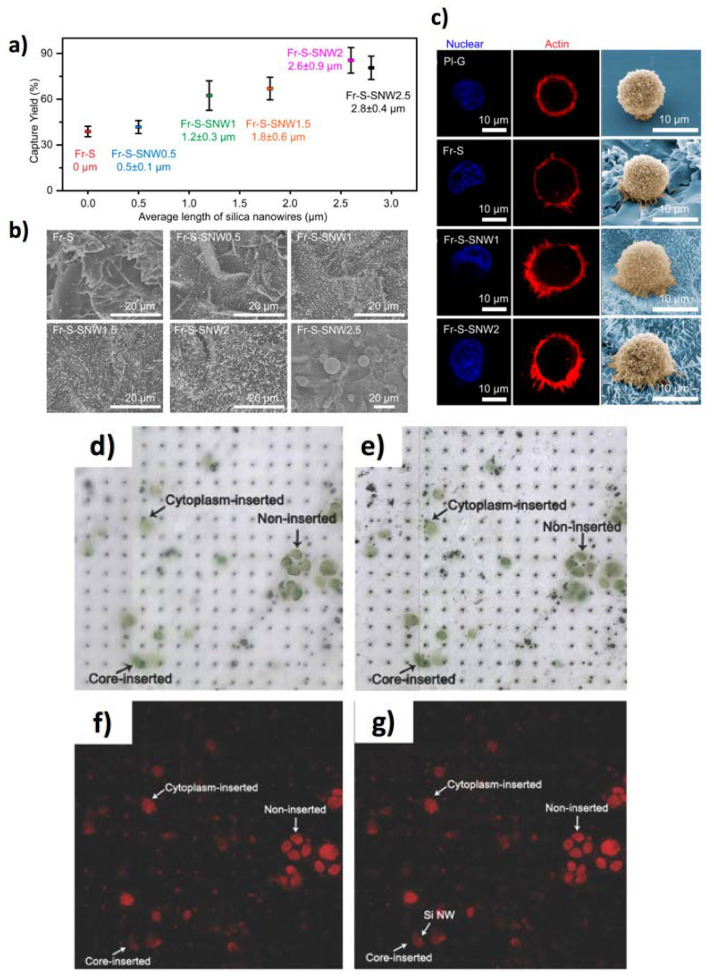
Single cell capture onto Si 1D structures. (**a**) NWs length-dependent triggered improvement of prostate cancer cell line (PC-3) capture. Fr-S stands for frosted slide, and Fr-S-SNW stands for SiO_2_ NWs-decorated slides at different NW lengths (0.5–2.5 µm). (**b**) Corresponding SEM images of the captured cells. (**c**) Immunofluorescence images staining (at the left and the centre) by nuclei DAPI and actin TRITC-phalloidine staining, and SEM images (at the right) of the captured PC-3 cells. Reprinted with permission from ref. [56] Copyright (2018) American Chemical Society. (**d**,**e**) Optical images, and (**f**,**g**) fluorescent microscopy images of Chlamy cells inkjet printed onto Si NWs, at 230 min (panels (**d**) and (**f**)), and at 300 min (panels (**e**) and (**g**)), showing core-inserted, cytoplasm-inserted, and non-inserted cells. Reproduced with permission from ref. [67] Copyright © 2016 by WILEY-VCH.

In another study, Feng et al. employed Si NWs (diameter of about 260 nm, density of 4 NWs·μm^−2^) to assess the cellular adhesion force modulated by the extracellular matrix [55]. They observed that the cell traction forces measured by a fibronectin covered Si NW array were higher than those obtained using a bare Si NW array. Si NWs prepared by chemical/ion etching [56,57,58,59,60] or their conjugates with thermo-responsive polymers [61,62] have found applications for the capture of tumour cells with extremely high yields (higher than 80%). Remarkably, Cui et al. [56] demonstrated a wet-chemistry in-situ growth of silica NWs functionalized with epithelial cell adhesion antibody (anti-EpCAM) to bind circulating tumour cells, achieving a binding efficiency up to 85.4 ± 8.3% for prostate cancer cell line (PC-3). Importantly, such functionalized silica NWs enhanced the capture efficiency of PC-3 more than 70% with respect to the corresponding flat surface [63] (Figure 3a–c). Patterning biological materials on solid surfaces is a convenient strategy to realize customizable devices for single-cell analysis. Among them, inkjet printing allows direct material deposition onto solid surfaces and is compatible with biomolecules and bioinks [64,65,66]. This method has been employed also for the insertion of living cells into vertically aligned Si NWs fabricated by deep reactive-ion etching [67]. Living Chlamy cells were printed in experimental conditions that did not compromise cell vitality, obtaining a suitable droplet speed (2–3 ms^−1^) that permitted to obtain a penetration force of the order of 6–7 × 10^−7^ N into the NWs. The misalignment error was reduced down to 7 μm, highlighting the very good accuracy of the cell-ink deposition onto the Si NW arrays (Figure 3d,e), observing that NWs can be inserted within different cellular zones, as reported in Figure 3f,g. 

From this section, the preeminent role of silicon-based 1D nanomaterials as key players within the fields of cellular stimulation, cell capture, sensors and drug delivery is clear, given their outstanding physico-chemical, electrical, and mechanical properties. 

## 4. TiO_2_-Based 1D Materials

Titanium dioxide (TiO_2_)-based 1D materials have high chemical stability, low-cost [68] and desirable chemical and physical properties [69,70,71]. In particular, TiO_2_ [72] has been widely employed in the fabrication of bone implants. The synthesis of TiO_2_ on Ti surfaces is typically carried out through an anodization method followed by heat treatment [73] An alternative method is constituted by the hydrothermal approach, in which Ti precursors, as Ti(IV) isopropoxide, are hydrolysed. The resulting products are dried and calcined to obtain TiO_2_ materials [74].

### 4.1. Applications in Bone Regeneration

TiO_2_ NTs are known to facilitate osteoblasts adhesion and propagation [75,76,77] via attraction between the osteoblasts and the Ti surface, onto which positively charged cellular adhesion proteins (such as fibronectin) are physisorbed [78]. Importantly, the size of the NTs is fundamental in eliciting the cellular reply, ultimately determining the cell fate, for example, of hMSCs, as reported in the following studies. Oh et al. [79] reported that NTs (30 nm in diameter) facilitated the attachment of hMSCs, whereas larger nanotubes (diameter in the range 70–100 nm) lead to higher cytoskeletal stress, ending up to hMSCs differentiating to osteoblasts. Sjöström et al. leveraged amphiphilic diblock-copolymers to prepare reverse micelles-based masks for anodizing of Ti surfaces [80], obtaining TiO_2_ NPLs (diameters of 20−30 nm and 15 nm height) that promoted the osteogenic differentiation of hMSCs [80,81,82]. 

On the contrary, de Peppo et al. [83] showed that the NPLs of TiO_2_ with larger dimensions (diameter of about 200 nm) facilitated the differentiation of hMSCs to osteoblasts. Park et al. [84] studied the effect of TiO_2_ NTs of two diameters (15 nm and 100 nm) functionalized with bone morphogenetic protein-2 (BMP-2). Significantly, the covalent immobilization of BMP-2 avoided MSC apoptosis that occurred on the uncoated TiO_2_ NT surfaces. Again, the lateral size of the 1D materials played a key role. Chondrogenic differentiation was favoured on the 100 nm BMP-2-covered NTs, whereas the 15 nm NTs led to osteogenic differentiation. Similarly, Zhou et al. [85] employed micro-arc oxidation to realize microporous coatings on Ti surfaces to favour differentiation of bone rat marrow MSCs. They followed the expression of integrin β1, osteogenic-related osterix, and alkaline phosphatase, finding that both the MSC adhesion and the osteogenic differentiation improved by increasing the pore size. Interestingly, Li et al. [86] discovered, by using an in vivo rat model, that both bone morphogenetic Protein-2 and TiO_2_ NTs enhanced the preosteoblast adhesion and osseointegration in comparison to bare Ti surfaces. The authors justified this behaviour by the sustained release of human bone morphogenetic protein-2 (hBMP-2), in comparison to the hBMP-2/Ti surface, which showed a rapid release.

### 4.2. Tools for Bacterial Decontamination 

The engineering of antimicrobial Ti-based biointerfaces is a crucial challenge for bone implants. A possible solution is the loading of antibiotics or antimicrobial peptides [87] onto such scaffolds. However, this can create an evolutive pressure on bacteria to become resistant to them. Many reports have tackled this issue by the engineering of Ti and TiO_2_ 1D biointerfaces that synergistically facilitate osteogenesis and enhance the bactericidal functions, given the different response of soft stem cells vs. rigid bacterial cells towards 1D materials interfaces. The antimicrobial activity can be enhanced by TiO_2_-enhanced visible-light-induced photocatalysis reactions that produce toxic species for bacterial cells (see below).

#### 4.2.1. Biomechanics Effects

The effects of the TiO_2_ biointerface on the mechanics-induced cell membrane deformation has been thoroughly investigated by Liu et al. [88] They showed that TiO_2_ NRs, produced by a two-step hydrothermal approach, modified the cell morphology and the nuclear shape of MCF-7 cells. In particular, the etching time during the hydrothermal synthesis tuned the size of the voids between TiO_2_ NRs. The higher the etching time, the larger the spaces between the NRs. The resulting voids induced a reversible mechanical deformation of the nuclei, regulating the pressure exerted by actin under the nucleus [88]. Similarly, Peng et al. [89] observed an increased *C3H10T1/2* cell adhesion along with reduced adhesion and colonization of *Staphylococcus epidermidis* (i.e., a pathogen associated with orthopaedic infections) in comparison with Ti surfaces. Again, this result was ascribed to the electrostatic effects due to the negative charge of the nanotubes that attracted osteoblasts and repelled the microbes. Biomechanics effects were leveraged also for inducing bacterial cells rupture without compromising cytocompatibility towards hMSCs. Hasan et al. [90] used reactive ion etching to yield NRs (height of about 1 μm and diameter in the 80 nm range), achieving maximal bactericidal efficiency (*E. coli*, *P. aeruginosa*, *M. smegmatis*, *S. aureus*). 

**Figure 4:**
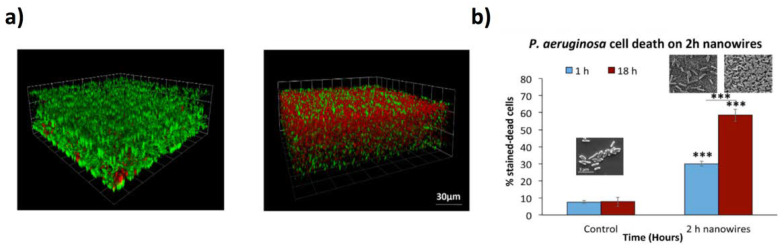
Antibacterial effects of TiO_2_ 1D materials. (**a**) Confocal images of *P. aeruginosa* on Ti (left) and TiO_2_ NW (right). In the picture, the healthy membranes are tracked in green (SYTO 9), whereas the compromised ones in red (propidium iodide). (**b**) The percentage of red stained *P. aeruginosa* cells on the NWs and control. The 18 h attachment produces more damages in comparison to 1 h attachment (see SEM images). The results were investigated by *t*-test, *** *p* < 0.001. Scale bars are reported in the figures. Reproduced from ref. [92] distributed under a Creative Commons Attribution 4.0 International License.

This mechanism was ascribed to the peculiar differences between bacterial and eukaryotic cells upon interacting with the 1D nanomaterials. Whereas the Young’s modulus of stem cells is in the range of 0.09 to 50 kPa, the bacterial cells show higher values, which result in more rigid cellular membrane and hence, easier rupture. Medina-Cruz et al. showed an intriguing synergic antibacterial activity by combining Ti NCs and Te NRs [91]. Ti NCs of different heights (150 nm and 300 nm long columns, tilted 20° and 40° from the normal, respectively) were synthesized by sputtering, showing antibacterial activity while maintaining the viability of human fetal osteoblastic cells. Upon functionalization with Te NRs, an enhancement of the antibacterial properties was observed, especially for the longest NCs. Although Te nanostructures themselves feature bactericidal effects, their combination with a nanocolumnar surface, which greatly reduces the bacteria adhesion as well, resulted in a synergetic enhanced bactericidal surface. In other reports, Tsimbouri et al. [92] proved that TiO_2_ NWs (average height of 1 μm and 25 nm in diameter) synthesized by a hydrothermal approach on Ti substrates supported the growth of osteogenic cells without stimulating osteoclast response, and reduced the viability of *P. Aeruginosa* bacteria (Figure 4a,b). In subsequent work, Bhadra et al. demonstrated that TiO_2_ can interact with bacterial cell walls (*P. aeruginosa* and *S. aureus*) leading to modest antibacterial behaviour [93]. Similarly to the paper of Tsimbouri et al. [92], the greater activity was ascribed to the gram-negative *P. aeruginosa* which had cell walls that were more easily deformed in comparison to the Gram-positive *S. aureus*. An effective bacteriostatic behaviour was demonstrated by Nataraj et al. on *S. aureus* by using a porcine skin model [94]. The authors found out that TiO_2_ NWs (about 100 nm in diameter) had higher antibacterial activity in comparison to TiO_2_ NPs (about 80 nm in diameter). It was possible to observe a concentration-dependent partial inhibition of *S. aureus* growth up to 4 wt % TiO_2_ NPs, whereas TiO_2_ NWs completely inhibited the growth. The reason for this different efficiency was explained by considering that, whereas NPs very easily aggregate, the NWs were better dispersed, leading to a higher anti-staphylococcal activity. 

#### 4.2.2. Photocatalysis

One-dimensional TiO_2_ materials have found many applications due to their photocatalytic properties [95], leading to the generation of hole and electron-hole pairs that, in turn, react and decompose the surrounding molecules (e.g., water and pollutants) [95]. Current research efforts also from our group, are focused on tuning the band gap energy and/or the specific nanomaterial surface area by altering the material shape [96], size and doping (nitrogen, metal, and carbon) [97], to favour the charge-transfer rate thereby increasing the photocatalytic activity of TiO_2_. Owing to these favourable photocatalytic properties, TiO_2_ based nanomaterials have been considered as high-efficiency antimicrobial agents since they can produce, under visible light, hydroxyl free radicals (OH) to destroy microbial systems. Some other reports have shown the antibacterial activity of 1D Ti-based materials, as for instance the electrospun zinc-doped TiO_2_ NFs [98], or photoactivated TiO_2_ coatings [99]. Recently, Munisparan et al. prepared TiO_2_ NWs by hydrothermal synthesis [100]. The anatase phase TiO_2_ NWs had remarkable photoinduced antibacterial activity towards Gram negative bacterial cells, such as *E. coli* and *K. pneumoniae*, whereas minimal inhibition was observed for the *P. mirabilis* and *P. aeruginosa*. 

### 4.3. Targeted Cancer Capture, Targeted Therapy, and Artificial Retinas Devices 

**Figure 5:**
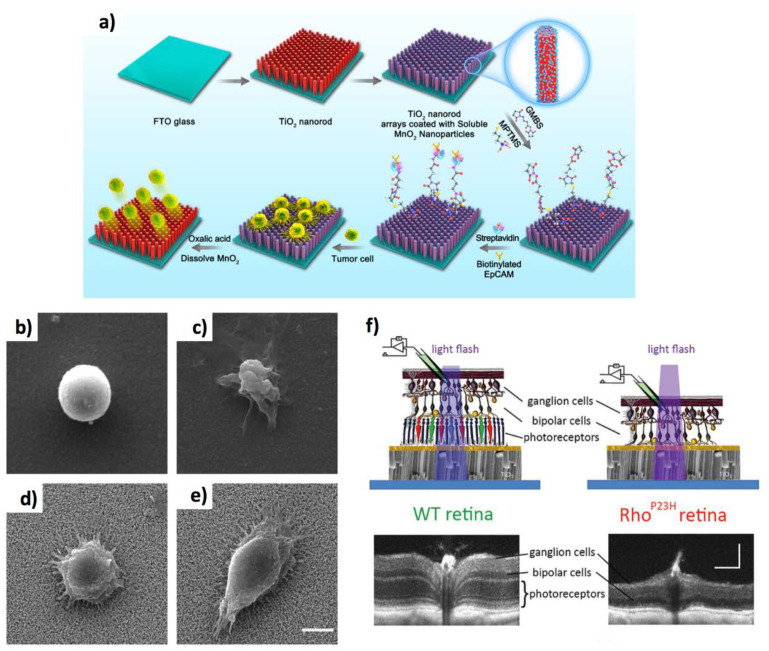
1D TiO_2_ materials for cellular capture and artificial retinas. (**a**) MCF-7 cells binding on surface functionalized TiO_2_ NRs. (**b**–**e**) SEM characterization of captured MCF-7 cells on different substrates (top left) FTO, (top right) MnO_2_/FTO, (bottom left) TiO_2_/FTO, and (bottom right) MnO_2_/TiO_2_/FTO. Scale bar: 5 μm. Reprinted with permission from ref. [101] Copyright (2018) American Chemical Society. (**f**) TiO_2_ NTs adhered with mouse retinas or bipolar cells (rhodopsin^P23H^ mouse retinas) trigger the retinal network activity. Reproduced from ref. [108] Copyright © 2018 by WILEY-VCH.

TiO_2_ 1D materials have been employed for other important applications. Li et al. [101] prepared surfaces based on TiO_2_ NRs by hydrothermal method and coated with transparent MnO_2_ NPs for the high-efficiency capture of CTCs (EpCAM-positive SW480 and MCF-7 cancer-cell lines—see Figure 5a–e). The surfaces were functionalized with 3-mercaptopropyl trimethoxysilan, N-maleimidobutyryloxysuccinimide ester and streptavidin (SA). In turn, the biotinylated anti-EpCAM was conjugated onto the SA-coated substrate. The EpCAM-positive cells were captured with very high efficiency (up to 92.9%), subsequently released at a very high release efficiency (89.9%) by treating the MnO_2_ NPs with oxalic acid to dissolve them from the platform, whilst not compromising cellular viability. Tian et al. [102] demonstrated that TiO_2_ NFs conjugated with various neurotherapeutic agents permit the efficient treatment of spinal cord injury. The neuroprotective activity of NF mediated compound delivery was ascribed to the reduced bioavailability or sustained drug release [103]. The TiO_2_ NWs administered together with rat MSCs facilitated the neuroprotection in diabetic rats under hyperthermia conditions [104] subjected to a blood–brain barrier breakdown. Whereas the administration of rat MSCs (1 × 10^6^) to diabetic rats slightly reduced the brain injury, TiO_2_ nanowired MSCs led to complete neuroprotection. The same group investigated the nanowired delivery of MSCs and cerebrolysin (a mixture of neurotrophic peptides) evaluating their effects on Alzheimer diseases in the rat model [105]. The TiO_2_-nanowired MSCs (10^6^ cells) in synergy with cerebrolysin increased the level of neprilysin, the key enzyme for beta amyloid peptide degradation. Interestingly, the combination of TiO_2_ NWs with MSCs and cerebrolysin was able to pass the blood–brain barrier. Research efforts have also explored some exciting applications in the field of neural stimulation and artificial retinas. TiO_2_ NTs doped with polyaniline were employed for nerve cell growth [106]. Polyaniline was deposited on TiO_2_ NTs by electrooxidation of aniline, improving TiO_2_ antibacterial activity, reducing the charge transfer resistance, and improving the anticorrosion properties. Indeed, S42 Schwann cells and PC12 cells on such scaffolds show improvement in proliferation, differentiation, and axonal growth. Tang et al. [107] fabricated an artificial photoreceptor at the interface with blind mouse retinas based on an oriented Au NPs-decorated rutile titania (Au-TiO_2_) NW array to induce light-triggered activities in the primary visual cortex in vivo, and pupillary light reflex in awake-behaving mice. Although promising, it is still not clear if this approach enables the space and time-dependent control for artificial retinas. In this regard, Ronzani et al. [108] have recently demonstrated that TiO NTs can greatly stimulate the functionality of healthy and blind retinas (Figure 5f). The authors placed films of anatase TiO_2_ NTs adhered with photoreceptors (mouse retinas) or with bipolar cells (rhodopsin P23H mouse retinas, which permits to model retinitis pigmentosa), observing that the 1D nanomaterials stimulated the retinal network up to the video rate frequencies (25 Hz), in response to micrometre sizes light spots. They showed the possibility to modulate the spot size, duration, and localization.

This section has allowed to describe the remarkable role of TiO_2_ based 1D materials systems within different applications such as cellular stimulation, cell capture, regenerative medicine, and bacterial photoinduced decontamination. Indeed, TiO_2_ based 1D materials can combine excellent mechanical properties, stability, photocatalytic activity, and possibility to be used as artificial photoreceptors.

## 5. Zinc Oxide-Based 1D Materials 

**Figure 6:**
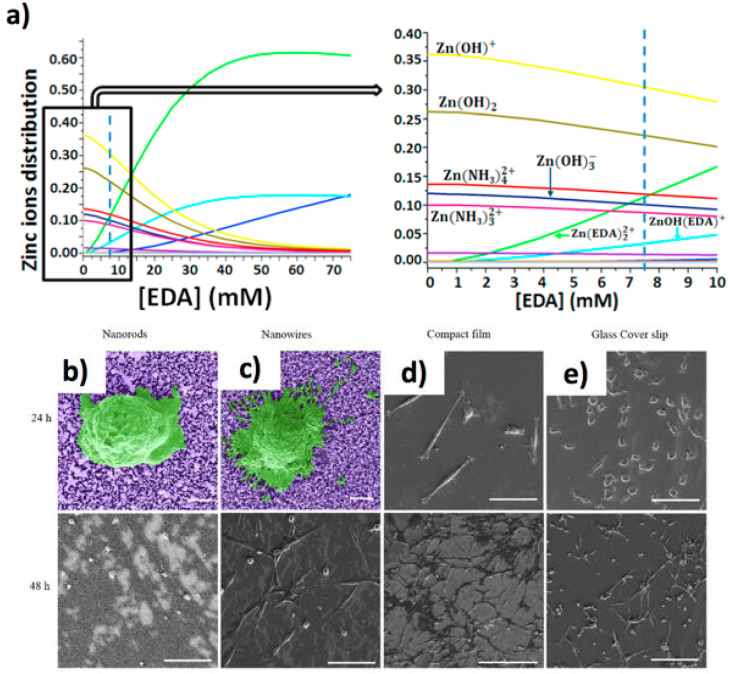

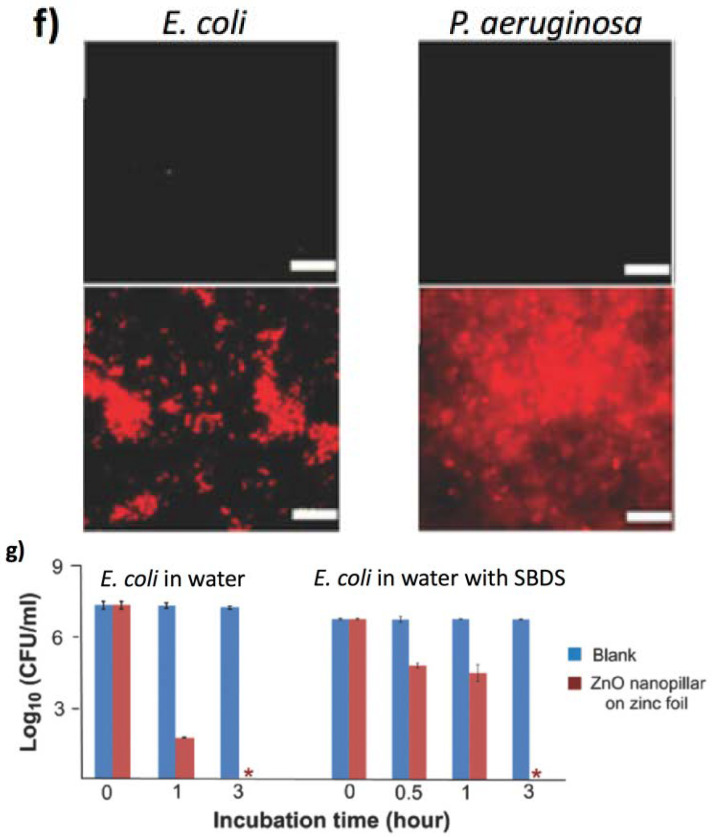
ZnO 1D materials for cellular lysis. (**a**) Control of zinc species by chemical speciation. The inset shows the effect of EDA within the concentration interval of 0–10 mM. Reprinted from ref. [16], Copyright (2018), with permission from Elsevier. Morphology of PC12 cells cultured onto ZnO nanointerfaces (after 24 and 48 h). ZnO NRs (**b**), ZnO NWs (**c**), ZnO film (**d**), and glass surface (**e**). Scale bars 1 μm. Reprinted from ref. [129] Copyright (2019), with permission from Elsevier. (**f**) Bactericidal activity of ZnO NPs grown onto glass slides. Fluorescence live/dead assay is shown for: (at the left) *E. coli*; (at the right) *S. Aureus*. Live cells are stained green, dead cells are stained in red. Scale bars are 20 µm. (**g**) Super microbicidal effect of ZnO NPs on Zn foil. The asterisk * indicates no colonies. The results are shown as mean ± S.D. of three experiments. Reproduced from ref. [137] Copyright ©2018 by WILEY-VCH.

ZnO 1D nanostructures are biocompatible [109], and can be easily functionalized given the presence of –OH groups at their surfaces [110]. The synthesis of ZnO can be carried out by vapour [111,112] or by low-temperature wet-chemistry approaches [83,113]. In contrast to chemical vapour approaches, which require high-temperatures, wet-chemistry methods can be carried out at low-temperatures (<90 °C) [112]. However, conventional wet synthetic methods might lead to poor reproducibility. To solve these issues, our group has developed a wet-chemistry fabrication strategy of ZnO NWs based on numerical modelling [16], revealing the optimal concentration of ethylenediamine, a zinc-binding molecule, to grow thin NWs at alkaline pHs (8.9–9) [114]. As a result, it was possible to control the precipitation of ZnO by favouring the synthesis of long ZnO NWs (>15 μm), and high aspect ratio (>200) (Figure 6a). 

### 5.1. Toxicity Issues: A Cell-Dependent Relation?

In general, ZnO NW based biointerfaces have cell-specific viability/toxicity which is partially dependent on the leakage of Zn^2+^ ions in the culture medium, and on the different morphologies and geometries of the 1D structures. As a consequence, it remains challenging to give a unified view concerning the biological effects of 1D ZnO material. Indeed, many literature reports showed a quite complex scenario, which lacks a clear recapitulation. For example, ZnO 1D materials are safe to Hela cells [115]*,* human Caco-2 enterocytes [116], human dermal fibroblasts [117], and osteoblasts, as shown by Zong et al. [118], Lin et al. [119], and Park et al. [120]. These cells exhibited improved adhesion, proliferation, differentiation, and growth on ZnO nanoflowers. However, other reports evidence lack viability in the case of RSC96 Schwann cells [121], MCF7 and HaCaT cells [122], MRC5 cells [121], neonatal rat cardiomyocytes [123], mouse calvarial cells [124], and other cancer cells such as HepG2 and Caski [125,126]. Similar lack of viability was observed for murine macrophages and human monocyte macrophages, respectively due to mechanical stress leading to necrosis [127] and ZnO NW intracellular dissolution in acidic pH [128], respectively. As a remarkable example, Ghaffari et al. [129] found that ZnO dissociation in the cell culture media prompted the release of zinc ions that modified the adhesion and viability of neuron-like PC12 cells (Figure 6b–e), observing cytotoxicity. Similar negative effects were found by Wang et al. [130] on NG108-15 neuronal cells, HL-1 cardiac muscle cells, and neonatal rat cardiomyocytes. Recently, Raos et al. showed that ZnO NWs [131] promoted the growth of human neuronal cells if the NWs were short (<500 nm) and packed (>350 NWs/µm^2^). On the contrary, ZnO NWs of higher length and lower density inhibited neuronal adhesion. The herein complex effects of the ZnO based biointerfaces were tentatively explained by Wang et al. [123] by considering that ZnO 1D materials at high density inhibit cellular adhesion, resulting in the loss of viability for fast dividing cell lines [130], whereas the lack of adhesion could still be tolerated by primary cells [132]. Moreover, ZnO 1D materials can also remove a thin solution layer below the cells, simply because the cell culture media tends to fill the spaces between the NRs due to capillary effects [133,134] thus limiting the diffusion of nutrient and oxygen to the cell, and resulting in cell death. 

Such intriguing cell-dependent toxicity effects stimulated researches also in the field of antibacterial properties. Similarly to TiO_2_, ZnO materials have also been employed as an efficient photocatalyst and consequently as antimicrobial agents [135]. For instance, Okyay et al. [136] investigated the toxic effects of ZnO NRs against *B. subtilis* and *E. coli*, also finding no significant toxicity to fibroblast cells. The authors found that the main anti-microbial mechanisms of ZnO NRs could be explained as a result of the production of H_2_O_2_ and bacterial cell membrane disruption. On the contrary, ZnO NPLs (about 1 µm long, and 120 nm in diameter) coated Zn foils exerted superbactericidal effects on *E. coli* (ATCC8739) [137], resulting from the superoxide ions release from the ZnO coating (Figure 6f,g).

### 5.2. New Frontiers in Regenerative Medicine, Drug Delivery, and Neuromorphic Devices 

**Figure 7:**
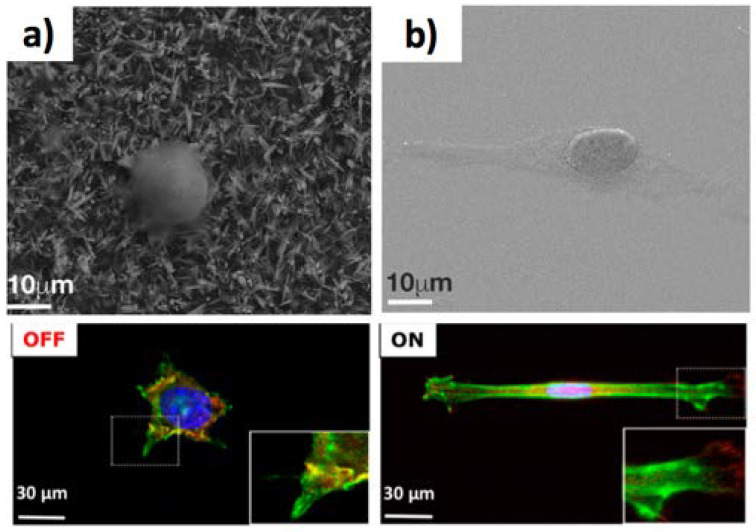

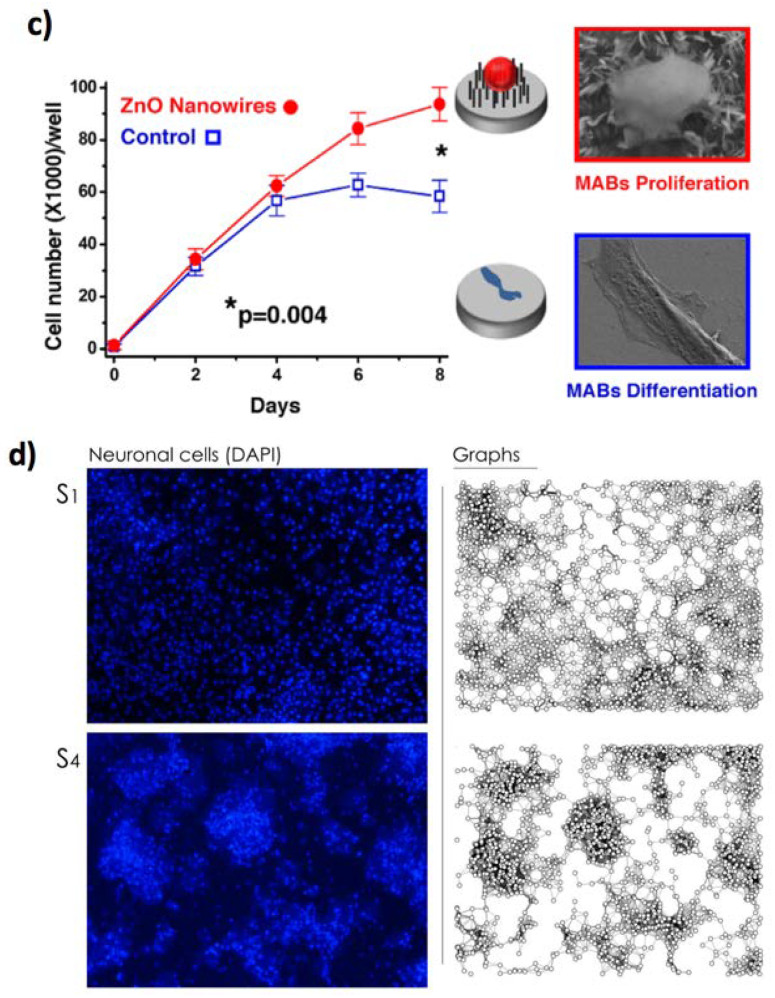

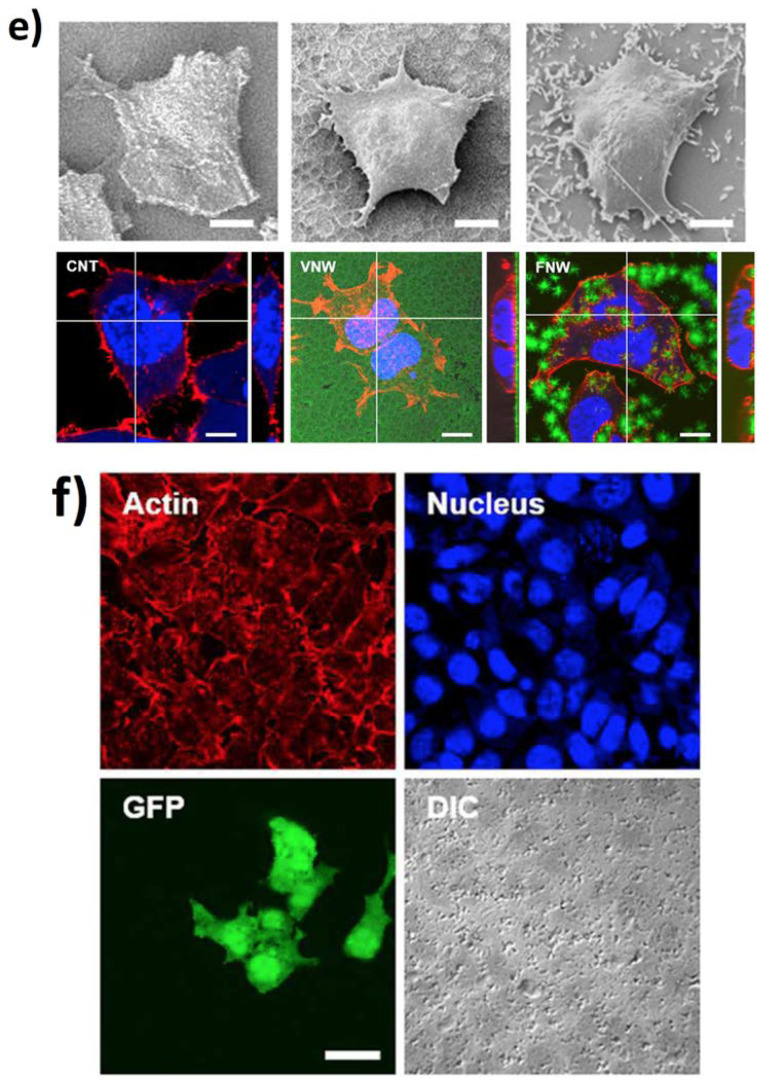
ZnO NWs for regenerative medicine and drug delivery. SEM and confocal microscopy images of the *MABs* (**a**) reduced spreading onto ZnO NWs, in comparison to (**b**) the control glass surface. Actin is stained in green and paxillin in red. (**c**) The MABs proliferation on glass (blue squares) or ZnO NWs (red circles). Reprinted with permission from ref. [132]. Copyright (2018) American Chemical Society. (**d**) The triggering of networks formed by hippocampal neurons onto ZnO NWs. On the left, the cells imaged via fluorescence. On the right, the wiring diagrams by a Waxman algorithm. S1 and S4 indicate two different samples. Reproduced from ref. [141], under the terms of the Creative Commons CC BY license. (**e**) Characterization via SEM images of the HEK293 cells cultivated on glass, VNW, and FNW. Cells cultivated on NW arrays for 48 h stained for cellular nuclei (blue) and cytoskeleton (red). (**f**) DNA-coated on FNW is intracellularly delivered to HEK293 cells leading to a GFP-expression construct. Reproduced from ref. [144] with permission from The Royal Society of Chemistry.

Regenerative medicine can be a fertile application for Zn-based 1D materials, triggered by the possibility to conjugate artificial environments that preserve cellular stemness with good antibacterial properties [138]. In particular, our group has proved that ZnO NWs can tune the differentiating abilities of mesoangioblasts (MABs) [132], a class of mesenchymal cells which are good candidates for applications in stem-cell therapy for muscular dystrophy [139]. Specifically, MABs cultured onto ZnO NWs showed a rounded morphology without lamellopodia during the cultivation, lacking the expression of myosin heavy chain, which is typical of differentiated myogenic cells (Figure 7a–c). The low cell adhesion strength was ascribed to the low density of focal adhesion complexes [140], resulting in the rounded morphology, as well as negligible NW penetration in accordance with theoretical models [27]. Such an NW biointerface was able to temporarily block the cellular differentiation. Besides, MABs recovered the differentiation capabilities upon regrowth on a standard culture glass. Very recently, the effect of the ZnO NR biointerface on the maintenance of cell stemness has been investigated at a molecular level by Kong et al. onto human adipose-derived stem cells (hADSCs) [20]. The authors demonstrated that the biointerface permitted cells to retain the stemness, whilst the Zn^2+^ release from ZnO NRs triggered the expression of KLF4 (Kruppel-like factor 4), a zinc-binding gene, which in turn triggered the expression of the transcription factors OCT4 and NANOG, promoting cellular stemness. ZnO NWs have also been applied as reconfigurable biointerface for neuronal cells. For instance, Onesto et al. [141] designed ZnO NW surfaces with variable NW density to direct hippocampal neurons assembly into clusters (Figure 7d). Interestingly, at specific fractal size and NW density, about 200 neurons are clustered, showing a small-world topology that recapitulates the natural structures of the cerebral cortex. As an intriguing application, ZnO NWs were also used to realize memristive devices for emulating the functions of biological synapses [142]. In this regard, Milano et al. showed an elegant neuromorphic system that emulated the Ca^2+^ dynamics of biological synapses [143] in the form of devices showing synaptic/resistive switching functionalities. They controlled the creation/rupture of Ag conductive path along the ZnO NW synthesized by CVD, permitting to mimic the synaptic Ca^2+^ dynamics. Another important application of ZnO NW biointerfaces is drug delivery. For example, Sharma et al. investigated the ZnO NW clusters mediated intracellular delivery of biomacromolecules [144]. In particular, they investigated cells cultivated on high-density ZnO NW as vertical (VNW), or fan-shaped (FNW) nanowires assemblies. Intriguingly, human embryonic kidney cells could survive and replicate without significant apoptosis, being the FNW wrapped with cells lamellipodia, allowing for the delivery of peptides or DNA molecules loaded on VNW and FNW (Figure 7e,f). In another study [145], Sharma et al. leveraged radially grown ZnO NWs onto poly-l-lactide microfibres as cancer vaccines, by loading them with the embryonic tumour antigen, inducing cellular immunity, for the in vivo inhibition of the tumour growth. Intriguingly, ZnO NWs on poly-l-lactide microfibres reduced the immune-suppressive TReg cells and favoured the recruiting of T cells into tumours. 

This section has permitted to highlight how ZnO-based 1D materials are starting to play an important role in engineering cellular interfaces, showing many points in common to TiO_2_-based systems given the cell-dependent toxicity effects due to Zn^2+^ release which also contribute to its antibacterial properties. Furthermore, key examples of applications of 1D ZnO biointerfaces in regenerative medicine, drug delivery, and neuromorphic devices are described.

## 6. Carbon Nanotubes

Carbon nanotubes (CNTs), hollow 1D nanostructures of rolled-up sheets of graphene, have outstanding physical and chemical characteristics which have made them key-players for photovoltaics [146,147], wearable electronics [148], drug delivery [149] and bioelectronics [150], showing differences and similarities to 2D-based counterparts [151]. They can be single- or multiwalled CNTs (SWCNTs and MWCNTs, respectively) depending on the number of graphene layers. CNTs can be synthesized by different approaches, such as arc discharge, laser ablation, and chemical vapour deposition (CVD) [152]. Importantly CNT synthesis can be compromised by impurities, such as carbonaceous materials and metals, that are removed by washes with acid solutions, and sonication in organic solvents [150]. Presently, the toxic effects of CNT are not fully understood, being influenced by many parameters, i.e., the amount of exposure, dose, dimensions, and surface functionalization [152]. Whereas pristine CNTs tend to easily aggregate, non-covalently functionalized CNTs (by polyethylene glycol or proteins) are not significantly cytotoxic [153].

### 6.1. CNTs-Powered Soft Robotics

**Figure 8:**
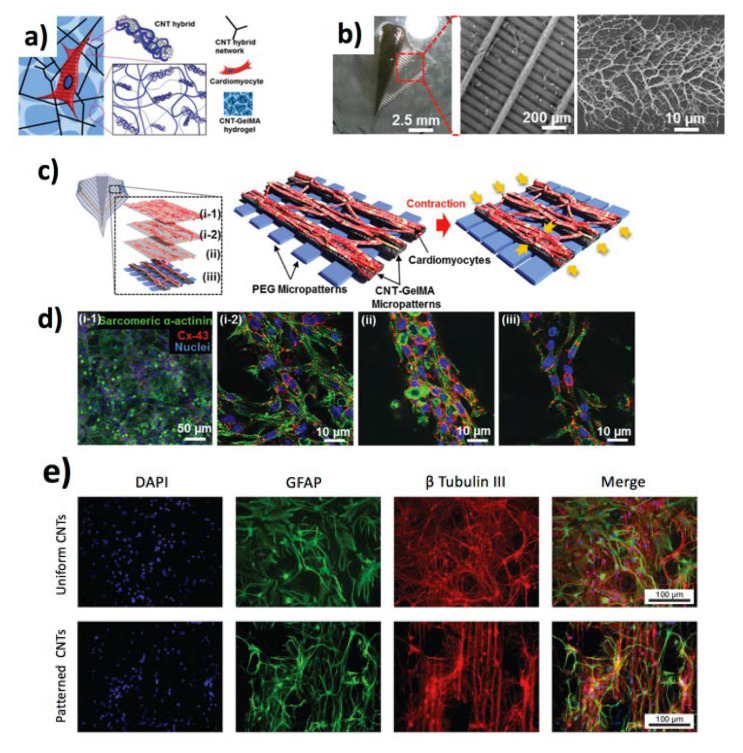

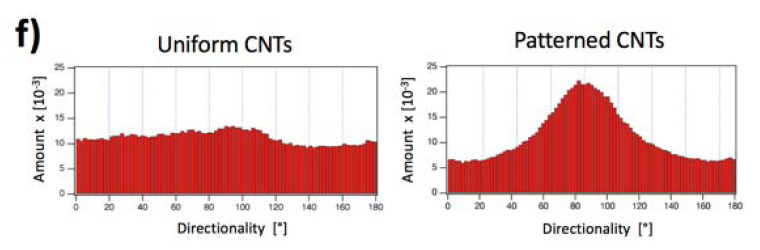
CNT applications in bioelectronics interfaces. (**a**) Electrically-driven soft robot composed of a PEG hydrogel substrate, covered by gelatin methacryloyl (GelMA) with dispersed CNTs (**b**) SEM characterization of the CNT–GelMA hydrogel. (**c**) The quasi-3D structure of the cardiac muscle tissue is constituted of four layers. (**d**) The corresponding confocal fluorescent images show different morphology in each of the systems. Reproduced with permission from ref. [156] Copyright © 2018 by John Wiley and Sons, Inc. Reprinted with permission from John Wiley and Sons, Inc. (**e**,**f**) The formation of a neural connection onto CNTs (top panels), and onto silicon decorated with patterned CNTs (15 µm pitch, 7.5 µm width) (bottom panels). DAPI staining for nuclei (in blue), *Glial fibrillary acidic protein* (GFAP) for astrocytes (in green), and β-tubulin III for neurons (red). At the left, uniformly distributed CNT carpet lead to homogeneous cellular distribution, at the right high directionality for the patterned CNTs. Reproduced with permission from ref. [158] Copyright © 2018 by WILEY-VCH.

CNTs are key materials for biointerfaces with electroactive cells [150], for instance, inducing the maturation of cardiomyocytes [154]. CNTs have also found applications in electrical potential registration, electrical stimulation of electrogenic cells [154], as well as in soft robotics. A very interesting example is shown by Goh et al. who prepared a bio-inspired hybrid CNT [155] powered by murine myoblasts C2C12 muscle cells. The authors prepared a poly(3,4-ethylenedioxythiophene) PEDOT/MWCNT-based hybrid muscle showing contraction and relaxation behaviour similar to the myotubes. Shin et al. [156] showed a bioinspired soft robotic system, with integrated self-actuating cardiac cells onto a hierarchically structured scaffold realized with PEG hydrogel substrate, and a composite of gelatin methacryloyl and CNTs, resulting in an actuation component for the soft robot (Figure 8a–d). In a similar report, Zhang et al. fabricated stretchable and transparent microelectrode arrays (MEAs), in which a network of CNTs was placed onto polydimethylsiloxane (PDMS) thin films, allowing for optical triggers, Ca^2+^ imaging and electrical signals registering on the rat motor cortex [152]. Recently, Scuratti et al. leveraged electrolyte-gated carbon nanotube transistors for real-time monitoring of cell adhesion and detachment [157]. Rago et al. [158] leveraged CNTs synthesized by CVD on a silicon surface for the electrical stimulation of primary neuronal cells extracted from rats. Interestingly, the spontaneous formation of a neuronal connection enhanced the electrical activity in comparison to glass, by means of the activation of short voltage pulses (Figure 8e,f).

### 6.2. CNTs-Based Tools for Cellular Stimulation, Cancer Therapy, and Drug Delivery

CNTs have found many applications in cancer therapy, scaffolds fabrication, and molecular delivery systems [159]. For instance, SWCNTs functionalized with phospholipids bearing polyethyleneglycol can be targeted into tumour cells [160]. Fadel et al. demonstrated that a CNT-polymer composite can be employed to cultivate cytotoxic T cells, allowing for applications in cancer immunotherapy [161]. In particular, they functionalized CNTs with antigens and polymer nanoparticles with magnetite and T-cell growth factor interleukin-2 (IL-2). Cancer cells were able to uptake the functionalized CNTs which, subsequently prevented them from dividing [162], by stabilizing the structures of protein microtubules against depolymerization [163]. This leads to mitotic spindles [164] and cell death. Beside these reports, MWCNTs have also been applied as a scaffold for bone regeneration if combined with biomaterials, such as bacterial cellulose, enhancing the scaffold mechanical characteristics, the support in osteoblast viability, adhesion, and proliferation in comparison to conventional culture substrates, as shown by the higher expression of vinculin and β1 integrin [165]. 

**Figure 9:**
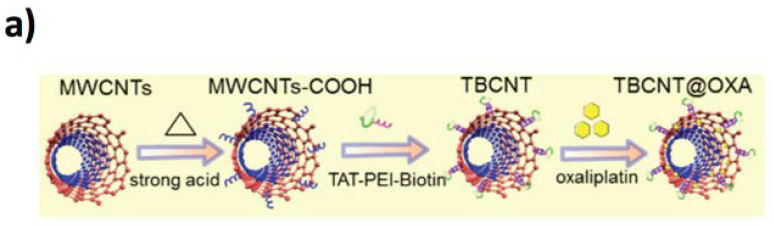

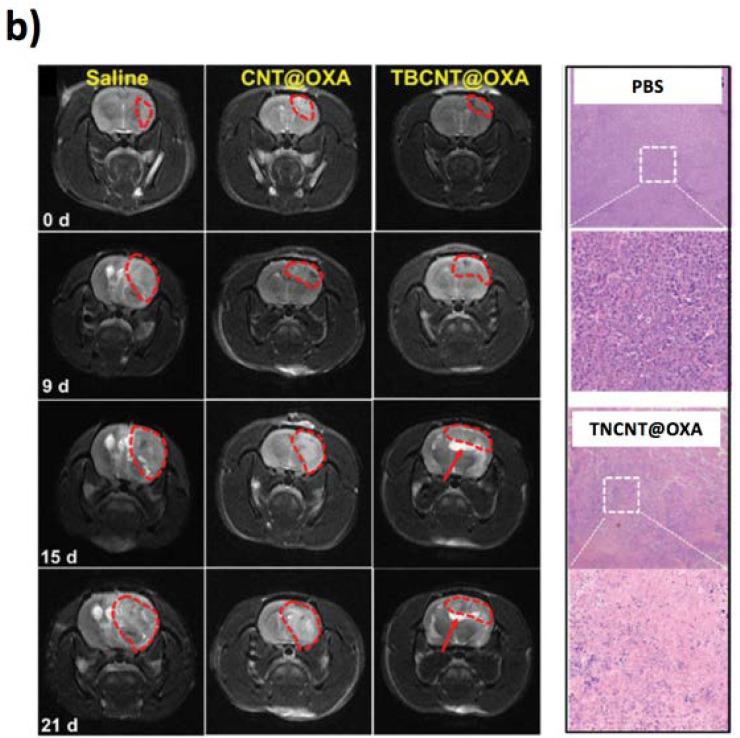
CNTs in cellular therapy. (**a**) MWCNT functionalization protocol with TAT-PEI-Biotin and OXA for glioma therapy to obtain the TBCNT@OXA system. (**b**) On the left, T2*-weighted pictures of tumours under treatment with control, CNT@OXA or TBCNT@OXA at various therapy days. On the right, images of haematoxylin and eosin staining of the tumours. Reproduced from Ref. [168] with permission from the Royal Society of Chemistry.

CNTs are also ideal drug delivery systems, given the possibility to functionalize their surface with biomolecules both covalently and non-covalently [166], facilitating the dispersibility of the CNT-drug complex in the biological fluids reaching the biological target. Gangrade et al. recently demonstrated an elegant drug delivery system containing an injectable CNT impregnated silk-based hydrogel [167]. In particular, they realized a composite made of silk protein and SWCTNs modified with doxorubicin permitting a sustained release within a 14-day study. In an interesting paper, You et al. showed a protocol of functionalization MWCNTs for orthotopic glioma therapy [168]. They used streptavidin-biotin coupling chemistry to functionalize the MWCNTs with the cell-penetrating peptide (TAT-PEI-Biotin) and oxaliplatin (OXA), obtaining the TBCNT@OXA system for in vivo targeting of gliomas (Figure 9a,b). 

This section has highlighted the fundamental role of CNTs within the field of bioelectronics, drug delivery and the recent applications in soft robotics and cellular therapy. The applications of CNTs in cellular interfaces are very well known. However, it is expected that the integration into artificial soft robotic devices might open up the way towards new researches.

## 7. 1D Polymeric Materials

The application of polymers as biomaterials has been intensely studied since the 1970s [169], opening up many relevant applications in life sciences, ranging from artificial organs to drug delivery systems [170,171]. Polymers are characterized by convenient stability and processability under mild conditions, adaptability to flexible substrates, as well as tuneable mechanical and electrical properties. The major methods for the preparation of structured polymeric materials include physical routes, i.e., electrospinning, mechanical stretching [172,173,174], as well as chemical routes, i.e., template-directed and template-free chemical growth, with diameters typically in the submicrometer scale [175]. Remarkably, tuning the biointerface between the biomaterial and cells is fundamental for developing biomedical applications [176,177]. The fundamental work of McMurray et al. [178] suggested a 1D polymeric based strategy for regenerating tissues. Specifically, they fabricated thermoplastic polycaprolactone (PCL) pits by hot embossing. A biointerface consisting of 120 nm pits 300 nm spaced ± 50 nm offset, leads to the differentiation of MSCs to osteoblasts. Intriguingly, by completely minimizing the offset, the MSCs maintained multipotency [178]. In a subsequent work, they improved the control of MSCs multipotency by tuning their adhesion [179].

### 7.1. Electrospun Polymers: From Natural to Synthetic Polymers

**Figure 10:**
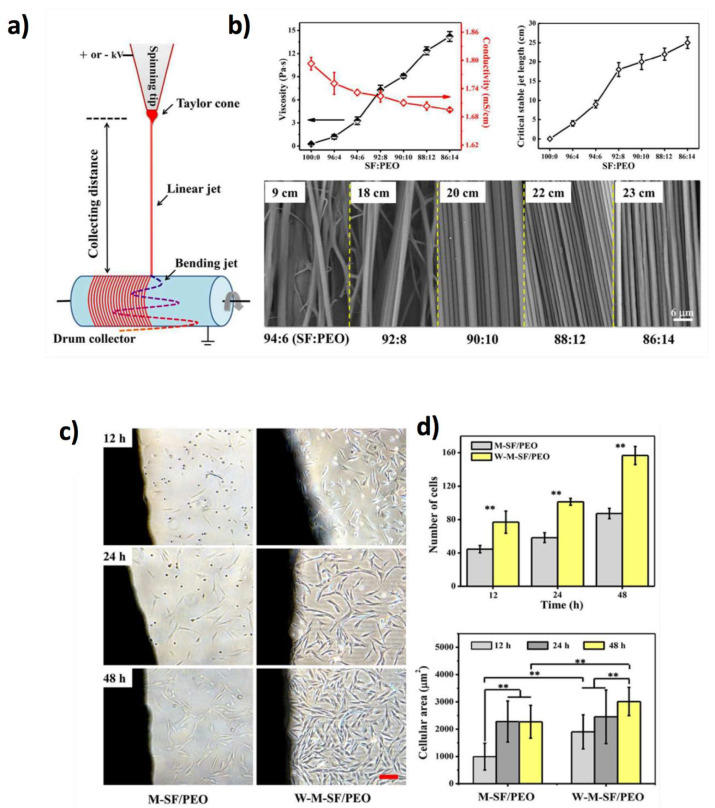

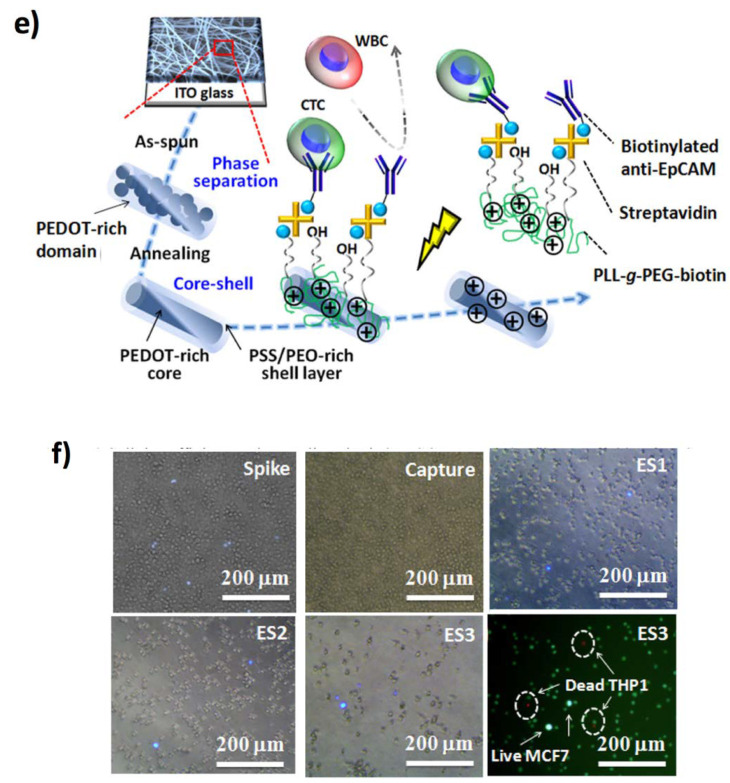
1D polymeric materials for cellular cultivation. (**a**) Stable jet electrospinning for fabricating polymer fibres. (**b**) At the top, effects of poly(ethylene oxide) (PEO) contents on physicochemical features and critical jet lengths of the SF/PEO inks. At the bottom, SEM characterization of the aligned SF/PEO fibres collected at stable jet lengths. (**c**) The iPS-MSCs morphology on the fibre scaffolds of M-SF/PEO and W-M-SF/PEO for 12, 24 and 48 h. Scale bars represent 100 µm. (**d**) At the top, quantification of cellular attachment and cellular dimension around the fibre scaffolds (*n* = 6). * *p* < 0.05, ** *p* < 0.01. Reproduced from ref. [184] with permission of Royal Society of Chemistry in the format Journal/magazine via Copyright Clearance Center. (**e**) Preparation of PEO/PEDOT:PSS nanofibre mats for the cell capture and release; (**f**) MCF7 cells (stained with Hoechst 33342) in THP1 cell suspension (without staining) under UV irradiation. The mixture cell suspension before the CTC purification (f top left, spike); after the CTC purification without electrical stimulation (ES) for cell releasing (f top centre); after ES1 (f top right); after ES2 (f bottom left); after ES3 (f-bottom-centre); cell viability after ES3 by live/dead staining assay (f-bottom-right). Calcein AM (green) for live cells; Eth-1 (red) for dead cells (*n* = 3). Reproduced with permission from ref. [206] Copyright year (2017) American Chemical Society.

Many researchers have recently demonstrated the outstanding potentiality of electrospinning for the engineering of 1D polymeric biointerfaces from natural and synthetic polymers [180]. Among the biopolymers that can favour cell adhesion, collagen is a very convenient choice, since it can be easily obtained from natural sources and at low cost, being the most abundant protein in the animal kingdom, where it plays the role of the major component of extracellular matrices [181] and can be used for cells immobilization [182]. However, it is difficult to obtain oriented 1D polymeric structures that can enhance oriented cellular growth and attachment. In this regard, Liu et al. fabricated collagen-doped dextran fibres to produce collagen fibres (between 5 and 20 µm in diameter) onto solid supports [183]. After treatment in aqueous solution to rehydrate the collagen, the dextran dissolved, producing a network of collagen fibres on the surface, which, in turn, directed the differentiation of C2C12 myoblasts. Silk fibroin (SF) is another biopolymer which has been fabricated into 1D fibres by jet electrospinning to mimic the ultrastructure of the extracellular matrix [184]. Highly aligned high-strength SF fibres ranging from 1.2 to 2.2 μm, were fabricated by adding poly(ethylene oxide) (PEO) to control the electrospinning process, leading to the alignment of pluripotent stem cells and the SF fibre mats (Figure 10a–d). 

Many recent approaches have shown the potentiality of electrospinning synthetic polymer fibres for the realization of polymer NFs resulting in scaffolds usable for cell culture [185]. Remarkably, polymer NFs can be loaded with additional components (drugs, nanoparticles, etc.) to exert further functions. In the following, some examples of emerging 1D-based polymeric materials and their relevant applications are reported. Among synthetic polymers, PCL is the most investigated, given its excellent processability, biocompatibility, and biodegradability. Herein, some PCL examples are reported. Sun et al. integrated PCL within a biomimetic trilayered fibrous scaffold, constituted by PCL fibres as an outer layer, fibres of PCL and polyurethane, and polyurethane fibres [186]. The fibres (diameter below 1 µm) were tuned by electrospinning. The obtained fibrous scaffold enhanced the growth and differentiation of primary rat MSCs mimicking the periosteum, the outer layer of bones. Electrospun PCL fibres have also been reported by Chen et al. [187] to realize hierarchically assembled structures from 2D-NFs mats of a controlled thickness into predefined 3D objects to interact with dermal fibroblasts. Recently, Omidinia-Anarkoli et al. [188] produced a strategy based on solvent-induced spinning to produce single PCL fibres. The solvent-induced spinning (i.e., absence of high voltage electrostatic field) allowed to investigate upon the influence of solvents employed for the formation of spin fibres (typical diameter around 10 µm), namely chloroform (CF), dimethylformamide (DMF), dichloromethane (DCM) and acetic acid (AA), and their combinations, to tune solubility and volatility, obtaining a morphological control of fibres as smooth (CF-AA, 50-50), grooved (CF-DMF, 75–25) and porous (DCM-DMSO, 50–50) fibres. The authors observed that porous and grooved fibres induced nuclear YAP translocation. 

Recently, the role of the alignment of PCL fibres (about 500 nm wide) produced from electro- or touch-spinning was found to be critical for directing the alignment, growth and differentiation of neural stem cells (NSCs) [189]. In particular, the higher crystallinity of the fibres from touch-spinning permitted to reproduce the extra-cellular environment of the early-stage neural differentiation, resulting in a bipolar elongation along the direction of the fibre. As mentioned above, other fibres forming composite polymeric materials have been investigated upon their biocompatibility, ability to induce cellular alignments, differentiation, and possible in vivo biodegradability. For example, Wang et al. reported that electrospun poly(3-hydroxybutyrate-co-3-hydroxyhexanoate) (PHBHHx) meshes favoured the MSCs differentiation to osteocytes [190]. 

An intriguing effect of surface potential imparted onto polyvinylidene fluoride (PVDF) fibres was observed by Szewczyk et al. for bone regeneration [191]. They tuned the surface potential of PVDF fibres by changing the voltage polarities during electrospinning, resulting in PVDF(+) and PVDF(−) fibres, observing higher osteoblast-like cell adhesion on the PVDF(−) scaffolds. Electrospun poly(lactic-co-glycolic acid) (PLGA) fibres allowed the orientation and differentiation of MSCs, as a combination of polymer molecule alignment and printed scaffold patterns [192]. Similarly, electrospun fibres of poly(d, l-lactide-co-glycolide) composite with collagen and hydroxyapatite (diameters around 320 nm) facilitated the osteodifferentiation of human adipose-derived stem spheroids [193]. The authors combined photolithography and electrospinning to realize a freestanding electrospun fibre mat to sustain the osteodifferentiation in comparison to the spheroids in the random substrate monolayers cultured on a random and patterned substrate. A similar osteogenic differentiation was observed on poly(l-lactic acid) (PLLA) submicrometer NF based scaffold onto which osteogenic growth peptide was immobilized [194]. Yap et al. [180] demonstrated the fabrication of poly(γ–glutamic acid)/β–tricalcium phosphate composites fibres (diameters ranging from 0.6 to 1.7 µm), permitting osteogenic differentiation. 

Among the polyesters materials, it is important to mention the poly (3-hydroxybutyrate-co-3-hydroxyvalerate) (PHBV) [195] which has been recently integrated as nanofibrous membranes with fibrinogen and bredigite (a magnesium silicate-based bone-mimicking bioceramic). The composite fibres have diameters comprised between 200 and 500 nm and favoured the proliferation of human fetal osteoblast unlike the fibres containing only PHBV and PHBV with fibrinogen. An interesting example of composite polymeric material is shown by Wu et al. [196] who prepared electrospun silk fibroin (SF) fibres (diameters ranging from 200 to 500 nm) scaffolds, which were surface modified by polydopamine, in turn, modified by grafting E7, a peptide allowing the capture of bone marrow MSCs and inducing via intracellular signalling pathways, osteogenic differentiation in a rat model. 

### 7.2. 1D Polymeric Biointerfaces for Cellular Electric Stimulation 

Polymeric NFs with electric conductive properties have been employed for engineering neuronal biointerfaces. As shown by Ferreira et al. [197] the dispersion of polypyrrole (PPY) fibres (diameter in the 100–1000 nm range) within biodegradable PCL/PLGA polymeric matrix permitted to increase the electrical conductivity up to 0.1 S·cm^−1^ [197]. In another example, Nekouian et al. [198] reported on the fabrication by electrospinning of PCL/PPY/MWCNTs fibrous structure for the electrical stimulation and differentiation of trabecular meshwork MSCs. The fibres had diameters in the range of 200–300 nm and were electrically conductive. The cells were seeded on PCL/PPY/MWCNTs scaffold and were stimulated by an electric field of 115 V/m, leading to a high expression of rhodopsin and peripherin genes, leading to potential applications in retinal regenerative medicine. 

Electrical stimulation on conductive polymer fibres also allows promoting osteogenic differentiation of bone MSCs on conductive fibres as proved by Jing et al. [199]. In particular, the authors fabricated conductive fibres by coating PPY onto electrospun PLLA fibres, obtaining conductive 1D materials with sub-micrometre sized diameters (960 ± 330 nm). Differently to the PLLA fibres, polypyrrole PPY/PLLA fibres triggered protein adsorption and mineralization nucleation. Upon the application of electrical stimulation, the fibres promoted further protein adsorption and mineral deposition. Bone MSCs cultured on these fibres differentiated to osteoblasts under electrical stimulation. The possible mechanisms involved in cellular stimulation under electrical stimulation are briefly described in the following. The electrical stimulation can enhance Ca^2+^ influx into cells via voltage-gated Ca^2+^ channel, favouring the osteogenic differentiation via Ca^2+^ ion signaling [200]. In addition, the formation of local electrical fields permits the regulation of the extracellular matrix protein synthesis and secretion, as well as the membrane protein localization, allowing the variation of the transmembrane potential [201]. Recently, poly(3-hexylthiophene) (P3HT) nano- and micro-fibres have been employed for triggering PC-12 neuronal stimulation and growth upon light stimuli [202]. 

### 7.3. Cancer Cells Capture, Bactericides Agents and Drug Delivery 

Along with regenerative medicine, 1D polymeric materials have shown potentialities as supports for capturing tumour cells, bactericidal devices, and drug delivery vehicles. The softness of 1D polymers with respect to inorganic materials affects their ability to maintain high cell capture efficiency [203]. As demonstrated by Sekine et al. [203], carboxylated PEDOT (PEDOT-COOH) nanodots bioconjugated with EpCAM antibodies increased the efficiency of specific tumour cells overexpressing EpCAM antigens, up to five times in comparison to smooth PEDOT-COOH films. Similar high efficiency in EpCAM expressing breast tumour cell capturing was obtained by Liu et al. [204] that used soft poly polystyrene (PS) NT substrates. The authors observed an 80% cell capture efficiency on PS NT substrate, significantly higher than flat PS and rigid Si NW-based devices. PEDOT-based micro/nanorod arrays have also been processed as a 3D bioelectronic interface, by using a combination of chemical oxidative polymerization and modified PDMS transfer printing [205] to produce a device capturing EpCam-positive cancer cells at 90% efficiency. PEDOT electrospun fibres mats have been demonstrated as powerful tumour cell capturing devices [206] based on the ordered deposition of biotinylated poly-l-lysine (PLL) PEG conjugate, SA, and anti-EpCAM-biotin. An electrically triggered detachment of the captured cells was achieved by applying cyclovoltammetric sweeps, reaching efficiencies as high as 87% (Figure 10e,f).

### 7.4. Antibacterial 1D Polymeric Biointerfaces

Polymeric 1D materials have also been employed as surfaces with intrinsic bactericidal activity and for drug sustained release, without any external physical activation. Notably, nature already employs “needle-based” antibacterial systems like the bactericidal cicada and dragonfly wing [207], by combining the strong adhesion and the mechanical shear forces between NPLs and bacteria. Mimicking natural systems, poly(methyl methacrylate) NPLs (spaced between 130 and 380 nm) were able to destroy Gram-negative *E. coli* cells [208]. Smaller spaced NPLs exhibited higher bactericidal activity, possibly because of on such surfaces, the bacteria are at contact with more NPLs, thereby experiencing higher stresses in more contact points. Polymer-based NWs can be leveraged for delivering drugs and biomolecules into cells, as demonstrated by the pivotal paper by Fox and coworkers [209] who carried out a drug/reagent non-sequential localization approach based on nanoimprint lithography to realize PCL NWs on which 3T3 fibroblast cells exhibited excellent adhesion.

**Figure 11:**
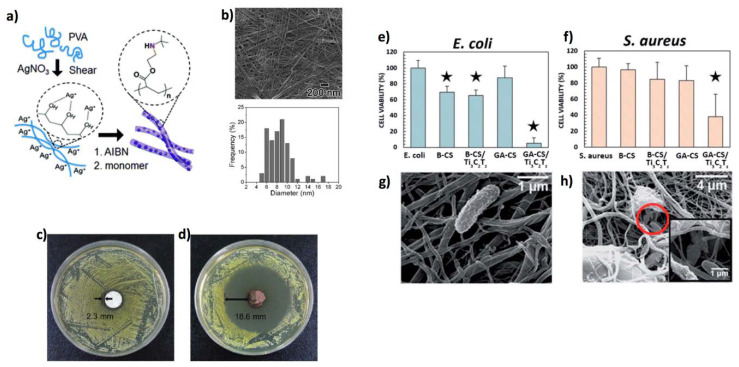
1D polymeric materials for bacterial decontamination. (**a**) Synthesis of Ag NP-embedded PTBAM NFs. (**b**) At the top, FE-SEM image of the silver NP embedded PTBAM NFs. At the bottom, size characterization of the silver NPs. (**c**,**d**) areas of inhibition of silver sulfadiazine and silver/PTBAM nanofibre respectively by the Kirby-Bauer test on 13 mm sized dishes. Reprinted with permission from ref. [210] Copyright (2012) American Chemical Society. Effect of Ti_3_C_2_T_z_ nanoflakes composited with electrospun chitosan (CS) NFs on (**e**) *E. coli*, and (**f**) *S. aureus* vitality. B-X and GA-X are systems processed with NaOH and glutaraldehyde, respectively. SEM characterizations show (**g**) unaffected, and (**h**) compromised *E. coli* bacteria on the 0.75 wt % Ti_3_C_2_T_z_/CS fibre entangle. The star indicates samples which are significantly different from the control, *p* ≤ 0.05. Reproduced under a Creative Commons Attribution-Non Commercial 3.0 Licence from ref. [217]—Published by The Royal Society of Chemistry.

Accordingly, 1D polymeric materials can be loaded with active antibacterial substances to exert a synergic effect. For example, Song et al. [210] prepared poly[2-(tert-butylaminoethyl) methacrylate] (PTBAM) cationic polymer NFs by radical-mediated dispersion polymerization, and embedded them with 8 nm-sized Ag NPs to exert bactericidal effects (Figure 11a–d). Some examples report on the encapsulation of Ag NPs within organic polymers, such as polyacrylonitrile fibres [211] (diameter comprised between 270 and 400 nm), and electrospun cyclodextrin NFs [212] (diameter around 200 and 400 nm), onto which Ag NPs were synthesized (cyclodextrins acted as reducing agents). In addition to Ag NPs, many reports also show the possibility to load antibiotics and bioactive compounds on the fibres. As an example, Kuntzler et al. developed a protocol to produce bactericidal electrospun NFs (about 200 nm in diameter) containing chitosan/PEO blend and phenolic compounds [213]. In other reports, interesting antibacterial activities were observed in poly(caprolactone)/poly(vinyl alcohol) core-shell NFs loaded with Thyme extracts [214] in PCL fibres on which gentamicin was immobilized [215] and also in nylon polymeric NFs membranes impregnated with Texas sour orange juice [216]. These materials all had diameters around 200–400 nm. Finally, it is worth mentioning the finding of Mayerberger et al. [217] who encapsulated Ti_3_C_2_T_z_ (where T is a variable surface termination such as OH, F, O, Cl) nanoflakes into electrospun chitosan NFs (diameters spanning in the 230–280 nm range) by crosslinking with glutaraldehyde. The authors observed that a Ti_3_C_2_T_z_ concentration as little as 0.75 wt % can induce bactericidal effects on *E. coli* and *S. aureus* (Figure 11e–h). 

Polymeric 1D materials have a lot of intriguing advantages with respect to all the other classes of 1D materials, given the favourable processability, tuneable properties and ideally suited mechanical properties which make them the most versatile 1D materials systems. They are typically realized in the form of fibres systems produced by electrospinning methods at low cost and high speed.

## 8. DNA and Peptide 1D Materials

Biomacromolecules, such as DNA, proteins and their shorter counterparts, namely oligonucleotides and peptides respectively, have remarkable characteristics such as biocompatibility, molecular recognition capabilities, and possibility to be engineered at the nanoscale by mild and green approaches. In particular, the modification of surface features of synthetic 1D materials through biomolecule chemi- or physisorption represents a fundamental strategy for their applications in biochip and biosensors fabrication.

### 8.1. 1D DNA Materials

Among the biopolymers, DNA has attracted increasing attention in the 1D material field due to its specifically programmable molecular recognition, chemical stability, as well as thermal assembly/melting control [218]. The possibility to design DNA single strands to drive a self-assembly route to virtually obtain whatever shape and structure, e.g., DNA origami, tiles, nanocages and so on, makes it ideal for controlling the assembly of other nanostructures [218]. Such DNA supramolecular architectures can play the role of templates for the preparation of metallic or polymeric nanoelectronic circuits, and any other desired object and device requiring resolution at the nanoscale. Liu et al. have investigated DNA assembly in ordered nanostructures, such as nanotubes and nanogrids, as well as the possibility to employ DNA as a template to grow conductive inorganic NWs using a metallization protocol [219,220].

**Figure 12:**
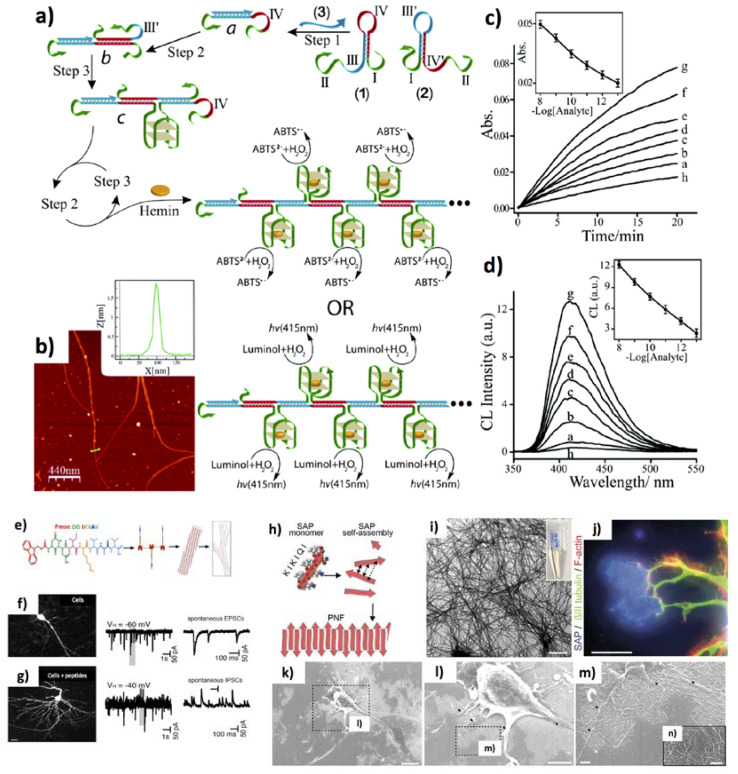
1D self-assembled DNA and peptides materials. (**a**) Target DNA recognition by two hairpins triggers the formation of DNA NWs. (**b**) AFM characterization and height analysis (inset) of the DNAzyme NWs. (**c**) Variation of ABTS^−^ absorbance as a function of time, at increasing DNA target concentrations in the range from 0 to 10 nM: (a) 0 M, (b) 10^−4^ nM, (c) 10^−3^ nM, (d) 10^−2^ nM, (e) 10^−1^ nM, (f) 1 nM, and (g) 10 nM. (**d**) Characterization via chemiluminescence spectra. Reprinted with permission from ref. [221] Copyright (2012) American Chemical Society. (**e**) The Fmoc-DDIKVAV peptide self-assembling in 1D structures by π-interactions of the Fmoc groups and the hydrogen bonds from the DDIKVAV peptide chains forming β-sheets, leading to a fibrous assembly. In (**f**,**g**) enhanced neuronal phenotype and synaptic connectivity of human embryonic stem cells implanted with peptide fibres into the ischemic brain. Scale bars are 20 µm. Reprinted from ref. [232] with permission from Elsevier. (**h**) Networks of self-assembling peptide NFs for neuronal cell adhesion. (**i**) TEM (scale bar is 600 nm), and (**j**) fluorescent microscopy (scale bar is 20 µm) images of peptide NFs forming plaques (blue) turning as adhesion points for nerve fibres (green). (**k**–**n**) SEM pictures of primary neurons peptide NFs (scale bars are 100 µm, 50 µm, 10 µm, and 5 µm, respectively), forming cellular protrusions (see the insets). Reproduced with permission from ref. [233] Copyright © 2019 by WILEY-VCH.

The applications of DNA as 1D nanomaterial are very promising, even though mainly related to biosensing field. An intriguing example was offered by Shimron et al., who developed an enzyme-free amplifying platform based on hemin/G-quadruplex DNAzyme NWs for detecting oligonucleotides [221]. This complex biomolecular system consists of functional nucleic acid hairpins containing the analyte-sensitive moiety, and the horseradish peroxidase-mimicking DNAzyme. Upon recognizing the target oligonucleotide, hemin/G-quadruplex DNAzyme wires are formed, along with the activation of DNAzyme that mimics the horseradish peroxidase. Then, the DNAzyme can play its role of functional element for the chemiluminescence-based detection, by catalyzing the oxidation of luminol or 2,2′-azinobis(3-ethylbenzothiazoline-6-sulfonic acid by H_2_O_2_. This biosensing platform was optimized for BRCA1 oncogene sensing down to of 10^−13^ M. Furthermore, He et al. have optimized a rapid DNA NWs assembly protocol at room temperature, and used it as a biosensor device for adenosine triphosphate detection [222]. A self-replicating catalysed hairpin assembly (SRCHA) was exploited to prepare DNA nanowires, starting from numerous target DNA replicas, that were produced with sticky ends allowing the double-stranded DNA single fibres to elongate and interact to induce the NW formation (Figure 12a–d).

### 8.2. 1D Peptide Materials

An alternative approach to chemi-/physisorption of biopolymers on solid supports is to prepare 1D materials by exploiting the biopolymers as fabrication building blocks. As an example of this strategy, Ryu et al. studied the assembly of diphenylalanine NWs [223]. Indeed, they showed the aniline vapour-mediated growth of peptide NWs by ageing an amorphous diphenylalanine film at temperatures above 100 °C. These NWs exhibit rigid and long shape (over 10 µm) suggesting high mechanical strength. Diphenylalanine dipeptides were also employed to assembly organic/inorganic hybrid NWs [224]. The same group, inspired by the process of self-arrangement of collagen fibres and carbonated hydroxyapatite in bone tissue, reported also on the synthesis of bone-like peptide/hydroxyapatite NWs. Such nanostructures were obtained through a mechanism mediated by coating the surface of the NWs with poly-dopamine. The central role of diphenylalanine NWs in peptide-based nanomaterials field was also demonstrated by their application in cellular studies and biosensors applications [225]. Indeed, Sasso et al. demonstrated that diphenylalanine NWs can be employed for biosensing, demonstrating that peptide-based NWs can sustain cancer cell adhesion and growth, and can be useful as an electrochemical platform, due to the easily available chemical modifications for conducting polymers immobilization.

As mentioned above, peptides can be exploited to increase the biocompatibility and cell internalization of 1D materials. In particular, the latter aspect was widely studied focusing on cell-penetrating peptides, short polycationic aminoacids sequences that mimic penetrating motifs present in translocation proteins. For instance, among these sequences, trans-activating transcriptional activator (TAT) has been shown to enhance the cellular uptake of Si NWs [226]. TAT protein-derived peptides were also employed to tune features of another kind of 1D nanomaterials, such as poly (ε-caprolactone) NFs for mesenchymal stem cell cultures to obtain soft support, where cell stemness is kept even after long-term passage culturing [227].

On the other hand, the same polymeric NFs were modified with different short peptides sequences to enhance the process of stem cell differentiation. In particular, Silantyeva et al. demonstrated that mouse embryonic stem cells showed higher neural differentiation on aligned NFs coated with the YIGSR peptide, which resulted faster than on laminin coating [228]. Intriguingly, the properties of functionalized peptide-based nanomaterials can be combined to build hierarchical structures, with increasing control on their assembly process. For example, collagen peptide NWs, able to assembly to form the peculiar collagen triple helix, were employed to that aim [229]. Properly combined with chemically modified Au NPs, collagen NWs led to an assembly process, based on peptides biomolecular recognition, which resulted in an extensive peptide/Au NP superlattice of cubic microcrystals. Further relevant applications of self-assembled peptide NFs have been demonstrated in the field of cell biology, as well. A paper from Harrington et al. demonstrated that branched peptide-amphiphiles can enhance bladder cells attachment to poly(glycolic acid) supports coated with peptides [230]. Lu et al. [231] demonstrated that bone-marrow-derived murine mast cell can easily adhere onto peptide NFs resulting in the inhibition of IgE–antigen-mediated degranulation but not in the non IgE–antigen-mediated degranulation. In a different report, Somaa et al. obtained “all-peptide fibre” scaffolds to mimic the IKVAV epitope, one of the brain’s extracellular protein (Figure 12e) [232]. The injection of embryonic stem cells together with such scaffolds reduced atrophy and triggered the rehabilitation of stroke in rat animal models. The systems containing embryonic stem cells on the scaffolds permitted to recover ischemia in rats at a significantly higher rate in comparison to cell- or scaffold-only implants (Figure 12f,g). In a breakthrough paper, Schilling et al. discovered three peptides from a library of 27 self-assembling peptides which can stimulate the growth and adhesion of neural cells belonging to primary mouse peripheral and central nervous systems [233]. The self-assembled peptides (SAPs) were injected into the site of an in vivo nerve lesion of a murine model, triggering the nerve reconstruction and recovering the bristle movement (Figure 12h–n).

The literature examples of pure biomolecular-based 1D materials are still few, however it can be expected that, given the ideally high biocompatibility, these systems will play a key role in the future engineering of biointerfaces for cellular stimulation. Ultimately, the chemi-/physisorption or self-assembly of DNA and peptides respectively can enhance biocompatibility, cell internalization, and differentiation of 1D materials in biosensing and bioelectronics.

## 9. Conclusions, Challenges and Opportunities

In this review, we have shown that 1D materials constitute outstanding tools for investigating biological systems with high resolution, selectivity, and immense versatility.

As a general principle, cellular systems are extremely sensitive to material geometry-induced cues.

The geometry of the material at the interface with cells plays a big role. Indeed, 0D materials are mainly employed as nanodispersions in culture media. Differently, 1D and 2D materials have been engineered for exerting stimuli in solution phase as well as at solid interfaces, with the main aim to reproduce many cues of the extracellular matrix. In this scenario, 1D materials have the unique capability to recapitulate the extraordinary complexity of the extracellular matrix, resulting in customizable interfaces that can mimic different biologically relevant phenomena, such as electrical stimulation, molecular delivery, intracellular sensing and other ones that have been described in the review. Moreover, it is well known that the geometrical features of 1D materials can be tuned to achieve tailored stimuli-to-cellular systems. In particular, by confining the lateral size at <100 nm, the resulting 1D nanomaterials can be leveraged as ideal minimally invasive nanoprobes for sub-cellular scale investigation, whereas larger widths are more ideally suited as fibre-like systems for extracellular scaffolds, to induce precise cell fate. Also, the density per unit area plays a relevant role: low densities (1–10 per 100 μm^2^) enable membrane entrance and investigation, whereas higher densities are associated with lower extent penetration.

A critical aspect investigated in this review is the further possibility to shape new 1D materials, leading to different properties and, in turn, different emerging applications. Accordingly, it is useful to briefly highlight the reasons why the particular material composition can be considered as specifically suited for each final application, like those that we have herein analyzed (i.e., regenerative medicine, cellular stimulation, bacterial decontamination, cellular capture, drug delivery, sensors and soft robotics, bioelectronics). The solid platforms for regenerative medicine and cellular stimulation are typically engineered with the aim to induce mechanic or electric stimuli that can recreate the niche conditions where cells can grow, be stimulated, and proliferate. Most of the researches in this field have focused on silicon, TiO_2_ polymers, and more recently, ZnO and peptides 1D-materials given the easiness flexibility of the synthesis. In the field of cellular capture, the most important examples come from the classes of silicon, TiO_2_, and again polymer materials, by leveraging surface chemistry protocols to obtain functionalized interfaces, for instance by using specific antibodies that can specifically capture cells of certain types. An important family of applications is represented by the bacterial decontamination leveraging both life-inspired mechanical stimuli and photo-induced degradation of bacterial cells. Apart from the canonical examples of ZnO and TiO_2_ which have remarkable photocatalytic properties, an important number of applications is constituted by polymers fibres loaded with antibiotics molecules. A particularly important field of application is drug delivery to cells. The mechanism of intracellular delivery mediated by such materials is typically based on the membrane poration, so the possibility to engineer needle-like systems that can also load molecules to be internalized plays a critical role. The involved 1D materials demonstrating the higher number of applications in this field are silicon, CNTs, ZnO, and polymers. In the field of bioelectronics and soft robotics, most of the initial literature considers the utilization of semiconductor-based materials (e.g., silicon, CNTs) and more recently new approaches based on polymers and DNA.

We confide that this review may give a fresh and hopefully useful overview in the field of 1D materials, focusing on how each material can play different roles. Silicon is, of course, the most investigated system for cellular coupling into devices, due to its favourable electrical properties and its prone integration in electrical devices for the direct sub-cellular investigation and manipulation. Notably, the interest in the applications of 1D metal oxides is increasing, especially considering their biocompatibility and low cost. Whereas TiO_2_ is a well-known system for bone regeneration and favourable antibacterial properties, with applications in the field of medical practice, there is no clear picture with regard to ZnO-based materials. Differently to TiO_2_, the complete integration of ZnO in devices is still not demonstrated, and more research effort is therefore needed for this material, also considering the role mediated in the zinc-responsive genes in the activation of intracellular cascades. Despite these issues, the ZnO synthesis conditions can be tuned, allowing for the generation of biocompatible platforms, and making feasible its employment in applications like regenerative medicine. Single and multiwalled CNTs have been very often employed for neuronal stimulation and cellular regeneration, and more recently for drug delivery. The potential toxicity issues can be solved by functionalization strategies, allowing for better dispersion in aqueous media and an easier cell-coupling. In addition, 1D polymeric materials can be considered as ideal systems for cellular regeneration, given their outstanding processability under remarkably mild conditions (electrospinning), and their tuneable chemical, mechanical, and electrical features, which are similar to those of inorganic nanomaterials. Finally, DNA- and peptide-based 1D materials are emerging as ideal biointerfaces for neuronal systems, owing to their intrinsic ability to modulate the cellular response in a biologically specific manner.

In our opinion, there are three major research topics currently under intense investigation that can boost the applications of the 1D materials in the world of biointerfaces. The first deals with the new approaches for the synthesis of the 1D materials. Many studies mainly employed silicon-based 1D structures produced by techniques deriving from the microelectronic industry. The trend is to produce 1D materials without the need of cleanrooms facilities and expensive patterning tools, featured with important properties such as flexibility, adaptability to soft surfaces, and better matching the mechanical properties of living cells. This trend is evident from the historical development of the field, which started with the development of silicon-based materials and is more recently evolving towards polymeric and biomolecular 1D materials assembly. The second point deals with the emerging range of achievable cellular stimuli provided by 1D materials since, in literature, the attention has been mainly limited to electronics and biomechanics. Only recently, researches have shown that there is room for a biochemical understanding of the cell behaviour at the interface with 1D materials, highlighting the importance of the role of the different 1D materials in triggering specific effects on the cellular behaviour, both from physical and chemical points of view. Finally, there are several possible widening applications of specific 1D materials-based platforms. Apart from the reported utilization in bioelectronics, 1D materials can tackle many issues of drug delivery, given their ability in modulating and perturbing the adhesion of the cellular membrane, and they are starting to become key players for cutting-edge applications such as bactericidal surfaces as well as cell supporting scaffolds for regenerative medicine and neuronal stimulation. In this regard, polymeric materials of natural and synthetic origin are receiving growing attention, also for the low cost and scalable approach of their synthesis. PCL-based fibres permit the realization of biomedical devices. Indeed, some of them are already on the market for regenerative purposes (e.g., Nanofibre Solution^TM^). The PCL-fibres can be considered as a valid approach in comparison to those produced from natural scaffolds (e.g., Biomimesys^®^), in terms of cost and stability.

In conclusion, this review has shown how 1D materials can be considered as general platforms for in-depth investigations in cell biology and medicine, allowing the discovery of new transduction pathways and intracellular responses which open the way to future approaches for drug delivery, regenerative medicine, and cellular stimulation. Based on the understanding of the specific cell–material interaction mechanism, it will be possible to integrate these materials in soft tissue-like devices, which can be directly applied in vivo. These challenges will be solved only by an efficient collaboration between material scientists and life scientists to establish a truly multidisciplinary knowledge platform.
